# Histone H4 lysine 20 acetylation is associated with gene repression in human cells

**DOI:** 10.1038/srep24318

**Published:** 2016-04-11

**Authors:** Jun-Ya Kaimori, Kazumitsu Maehara, Yoko Hayashi-Takanaka, Akihito Harada, Masafumi Fukuda, Satoko Yamamoto, Naotsugu Ichimaru, Takashi Umehara, Shigeyuki Yokoyama, Ryo Matsuda, Tsuyoshi Ikura, Koji Nagao, Chikashi Obuse, Naohito Nozaki, Shiro Takahara, Toshifumi Takao, Yasuyuki Ohkawa, Hiroshi Kimura, Yoshitaka Isaka

**Affiliations:** 1 Department of Advanced Technology of Transplantation, Osaka University Graduate School of Medicine, 2-2 Yamadaoka, Suita, Osaka 565-0871, Japan; 2 Department of Nephrology, Osaka University Graduate School of Medicine, 2-2 Yamadaoka, Suita, Osaka 565-0871, Japan; 3 Division of Transcriptomics, Medical Institute of Bioregulation, Kyushu University, Fukuoka 812-8582, Japan; 4 Graduate School of Frontier Biosciences, Osaka University, 1-3 Yamadaoka, Suita, Osaka 565-0871, Japan; 5 Graduate School of Bioscience and Biotechnology, Tokyo Institute of Technology, 4259 Nagatsuta-cho, Midori-ku, Yokohama 226-8501, Japan; 6 CREST, JST, 4-1-8, Honcho, Kawaguchi, Saitama 332-0012, Japan; 7 Institute for Protein Research, Osaka University, Yamadaoka 3-2, Suita, Osaka 565-0871, Japan; 8 RIKEN Center for Life Science Technologies, 1-7-22 Suehiro-cho, Tsurumi-ku, Yokohama, Kanagawa 230-0045, Japan; 9 RIKEN Structural Biology Laboratory, 1-7-22 Suehiro-cho, Tsurumi-ku, Yokohama, Kanagawa 230-0045, Japan; 10 Radiation Biology Center, Kyoto University, Yoshidakonoe-cho, Sakyo-ku, Kyoto 606-8501, Japan; 11 Graduate School of Life Science, Hokkaido University, Kita 21 Nishi 12, Sapporo 001-0021, Japan; 12 MAB Institute Inc., Kita 21 Nishi 12, Sapporo 001-0021, Japan

## Abstract

Histone acetylation is generally associated with gene activation and chromatin decondensation. Recent mass spectrometry analysis has revealed that histone H4 lysine 20, a major methylation site, can also be acetylated. To understand the function of H4 lysine 20 acetylation (H4K20ac), we have developed a specific monoclonal antibody and performed ChIP-seq analysis using HeLa-S3 cells. H4K20ac was enriched around the transcription start sites (TSSs) of minimally expressed genes and in the gene body of expressed genes, in contrast to most histone acetylation being enriched around the TSSs of expressed genes. The distribution of H4K20ac showed little correlation with known histone modifications, including histone H3 methylations. A motif search in H4K20ac-enriched sequences, together with transcription factor binding profiles based on ENCODE ChIP-seq data, revealed that most transcription activators are excluded from H4K20ac-enriched genes and a transcription repressor NRSF/REST co-localized with H4K20ac. These results suggest that H4K20ac is a unique acetylation mark associated with gene repression.

Post-translational histone modifications, such as lysine acetylation and methylation, play important roles in the epigenetic regulation of gene expression through chromatin structure changes. Histone acetylation is generally associated with transcriptional activation by recruiting effector proteins that harbor acetyl-binding domains, and possibly also by neutralizing positive charges to weaken electrostatic histone–DNA interactions^1^. Indeed, histone H3 acetylated at lysine 9, 14, or 27 accumulates around transcription start sites (TSSs) and/or enhancers of transcriptionally active genes^2^. It has been shown that acetylation of different lysine residues in the N-terminal tail of H4 has distinct functions. Hyperacetylated H4, in which lysine 5, 8, 12, and 16 are all acetylated, is associated with highly active genes^3^, while lysine 16 acetylation is more abundantly distributed and has a specific role in cellular senescence^4^. Di-acetylation of H4 at lysine 5 and 12 is associated with newly-assembled chromatin. Unlike these residues, acetylation at H4 lysine 20 (H4K20ac) has not been characterized. This modification was initially found in yeast^5^ and plant (*Arabidopsis thaliana*)^6^, but recent mass spectrometry analyses using ^13^C isotope tracer have shown H4K20ac is also present in human HeLa cells and exhibits a fast turnover^7^. Here, we analyzed the distribution of H4K20ac using a specific monoclonal antibody. Unexpectedly, H4K20ac was enriched around TSSs of minimally expressed and silent genes, and also in the gene body of expressed genes. These results suggest that H4K20ac is a previously undescribed type of histone acetylation that is associated with gene repression.

## Results

### Detection of H4K20ac by mass-spectrometry

**Figure 1:**
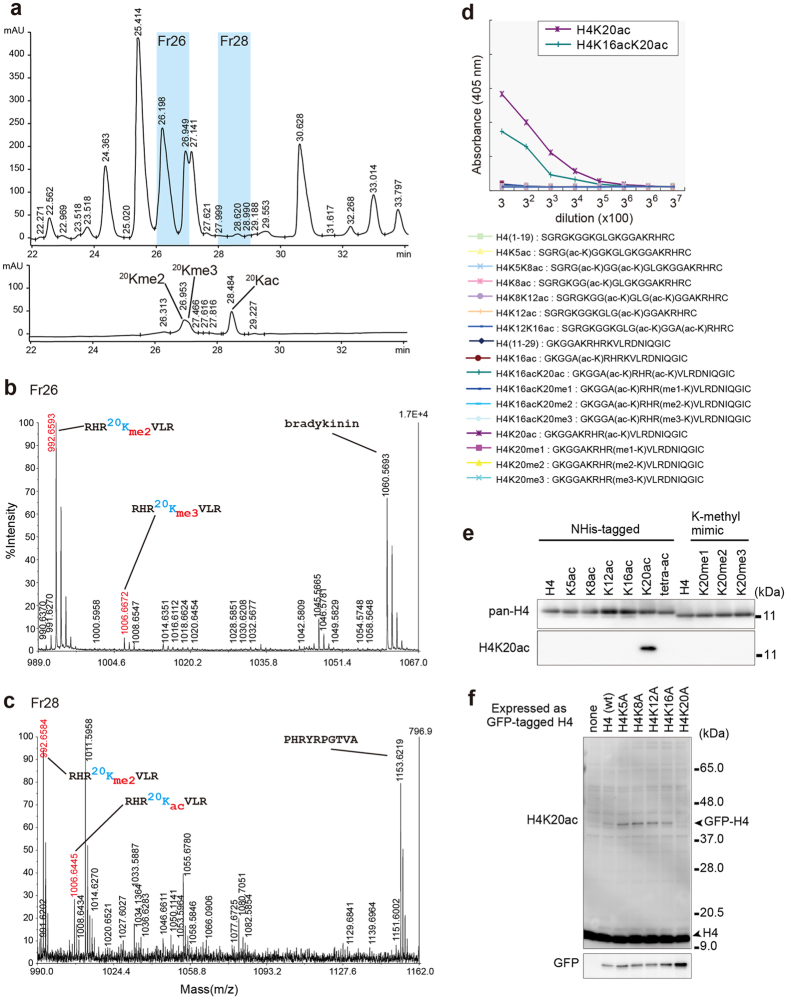
Detection of H4K20ac by specific antibody and mass spectroscopy. (**a–c**) Detection of endogenous H4K20ac by HPLC-mass spectroscopy. (**a**) HPLC profiles. Digested histone H4 prepared from HeLa cells (upper trace) and a mixture of synthetic peptides with the sequence RHR^20^KVLR (lower trace) are shown. (**b**,**c**) MALDI mass spectra. HPLC fractions Fr.26 (**b**) and Fr.28 (**c**) were analyzed. Accurate masses of the target peptides were determined by interpolation of the two internal calibrants: RHR^20^Kme2VLR (MH^+^: 992.6588) and bradykinin (MH^+^: 1060.5687) for Fr. 26; and RHR^20^Kme2VLR and PHRYRPGTVA (histone H3, 38–47; MH^+^: 1153.6225) for Fr. 28. The measurements were repeated five times to give the accurate masses of the target peptides as 1006.6695 ± 0.0026 (Fr. 26) and 1006.6434 ± 0.0018 (Fr. 28). Typical examples for identification of H4K20me3 (**b**) and H4K20ac (**c**) are shown. (**d**–**f**) Characterization of the H4K20ac-specific antibody. (**d**) Results from an ELISA using the H4K20ac-specific antibody. (**e**) Immunoblotting using recombinant H4 proteins harboring the indicated modifications^29^. tetra-ac: H4K5acK8acK12acK16ac. (**f**) Immunoblotting using transiently expressed GFP-tagged H4 harboring site-specific lysine to alanine substitutions. (**e,f**) The gel or immunoblotting pictures were cropped from original full length immunoblots (Supplementary Fig. S5). The gels were run under the same experimental conditions.

We first used mass spectrometry to examine whether the H4K20ac modification is present in our HeLa-S3 cells. Given that the H4K20ac abundance could be very low and that acetylated and trimethylated lysines have very similar masses, we employed a targeted approach by isolating H4 RHR^20^KVLR peptide from purified HeLa H4 using high-performance liquid chromatography (HPLC; Fig. 1a and Supplementary Fig. S1). RHR^20^KacVLR (measured mass: 1006.6434 ± 0.0018) was identified in fraction (Fr) 28, separated from RHR^20^Kme3VLR (measured mass: 1006.6695 ± 0.0026) in Fr 26 (Fig. 1b,c). This data demonstrates that H4K20ac does exist in HeLa-S3 cells, which is consistent with a recent report showing that H4K20ac is a modification generally found at low abundance, occupying about 0.3% of the total amount of acetylated lysines^7^.

### Generation of H4K20ac-specific antibody

To obtain insights into the function of H4K20 acetylation, we generated a mouse monoclonal antibody directed against H4K20ac. An enzyme-linked immunosorbent assay (ELISA) showed that this antibody reacted specifically with peptides containing acetylated K20 (Fig. 1d). The specificity was further confirmed by immunoblotting using recombinant H4 proteins containing specific modifications and point mutations. Among various H4 proteins harboring acetylation at different lysine residues and three levels of methylation-mimic modifications at K20, only H4 acetylated at K20 was recognized by the antibody (Fig. 1e). The antibody also reacted with GFP-H4 expressed in HeLa cells and with related mutants harboring lysine-to-alanine (KA) substitutions, the lone exception being the K20A mutation (Fig. 1f). These results indicate that this newly generated antibody is specific to H4K20ac.

### H4K20ac distribution by ChIP-seq

**Figure 2:**
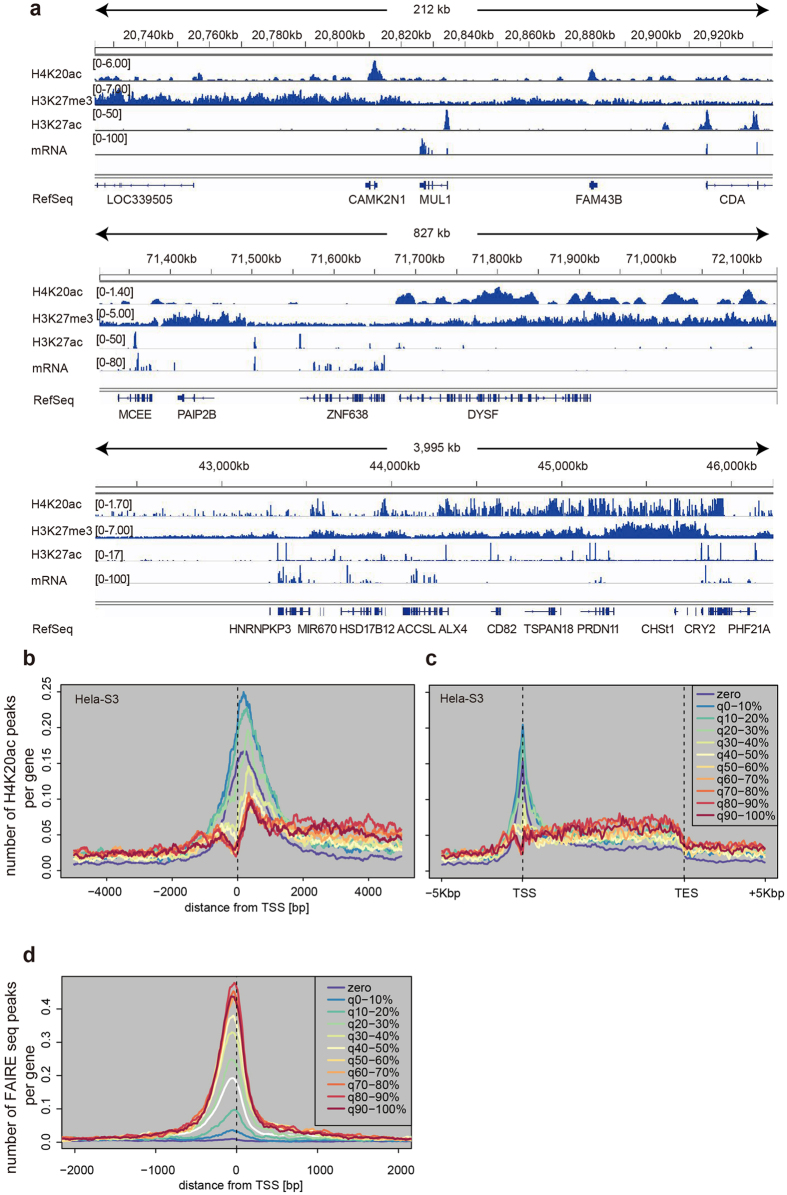
Distribution of H4K20ac in HeLa-S3 cells. ChIP-seq analysis was performed using the H4K20ac-specific antibody. (**a**) Distribution of H4K20ac, H3K27me3, H3K27ac, and mRNA at genome loci spanning 212 kb (top), 827 kb (middle), and 4 Mb (bottom). (**b,c**) Aggregation plots. Averaged peak counts of H4K20ac signals surrounding the transcription start site (TSS; **b**) and across the gene bodies (**c**) are indicated for 11 groups of genes, which were categorized based on their expression levels. (**d**) FAIRE-seq. Averaged peak counts of FAIRE-seq signals around the TSS are shown for the same 11 groups of H4K20ac-enriched genes, which were defined by the presence of H4K20ac MACS peaks within ±1 kb from TSSs.

To determine the H4K20ac localization in HeLa-S3 cells, we performed a ChIP-seq analysis using the H4K20ac-specific antibody (Fig. 2 and Supplementary Fig. S2-1a). When viewed using a genome browser together with mRNA-seq and ENCODE ChIP-seq data for H3K27me3 and H3K27ac (typical marks for repressed and active genes, respectively), H4K20ac signals appeared to be associated with gene loci that had low or no expression of mRNA, rather than with highly transcribed genes (Fig. 2a). For example, H4K20ac was enriched in CAMK2N1 and FAM43B loci but not in MUL1 and CDA loci, where H3K27ac and mRNA were observed (Fig. 2a, top). These gene loci were also devoid of H3K27me3, even though it was distributed broadly around these genes (Fig. 2a, top). Similar non-overlapping distributions between H4K20ac and H3K27ac or H3K27me3 were also observed in wider views, although the relationship between H4K20ac and H3K27me3 became less obvious at these resolutions (Fig. 2a, middle and bottom). These results suggest that H4K20ac is a unique acetylation that, unlike H3K27ac, is not associated with highly transcribed genes.

We then profiled the distribution of H4K20ac around the TSSs and along the gene body in gene sets classified by mRNA levels. Aggregation plots based on MACS peak call (Fig. 2b,c) showed H4K20ac was enriched around TSSs of minimally-expressed genes (q0–10, q10–20, and q20–30) compared with those of silent (zero) and highly-expressed genes. Similar patterns were observed when aggregation plots were drawn based on average signal intensity (Supplementary Fig. S2-1b,c), and when genes were categorized using the degree of H3K4me3 enrichment around TSSs (Supplementary Fig. S2-1d). The limited enrichment of H4K20ac in the TSSs of highly expressed genes could be due to lowered nucleosome density in those genes. However, this is unlikely because the enrichment was normalized using the input (see Methods section for details), and, unlike H4K20ac, H3K4me3 was enriched in highly expressed genes, as shown previously, indicating that nucleosomes are present in those genes (Supplementary Fig. S2-2a,b). H3K9me3 was slightly enriched in unexpressed genes, but did not show peaks around TSSs, suggesting that the enrichment around TSSs of minimally expressed genes is not a common feature for silent marks (Supplementary Fig. S2-2c,d).

In addition to accumulation around TSSs in minimally-expressed genes, a subtle accumulation of H4K20ac within gene bodies was also observed in highly-expressed genes (Fig. 2c and Supplementary Fig. S2–3a). The degree of H4K20ac enrichment in gene bodies was much lower than that of H3K36me3 (Supplementary Fig. S2–3b). These results suggest that the distribution of H4K20ac differs both from general histone acetylation marks, which are associated with active genes^2^, and from repressive marks. To examine the reproducibility of H4K20ac ChIP-seq in different conditions and in another cell type, we performed ChIP-seq analyses using HeLa cells that were treated with interferon γ and human smooth muscle cells (hSMC). A scatter plot analysis indicated that the distribution of H4K20ac in interferon-treated cells were highly similar to that of the untreated cells (Pearson’s correlation coefficient 0.958; Supplementary Fig. S2–4). The enrichment of H4K20ac in minimally expressed genes was also observed in hSMC (Supplementary Fig. S2–5). These data suggest that H4K20ac is robustly and generally associated with transcriptional repression in human somatic cells.

### H4K20ac-enriched regions are not highly accessible

To assess whether chromatin is in an open state in H4K20ac-enriched genes, we analyzed FAIRE-seq data produced by the ENCODE project^8^,^9^. As shown previously^8^, this analysis mainly detects TSSs of highly-expressed genes (Fig. 2d). The H4K20ac-enriched genes were in the range of genes expressed at much lower levels (between the 10–20% and 20–30% deciles of genes). A genome-wide scatter plot analysis also showed little correlation between H4K20ac and FAIR (Supplementary Fig. S2–6). Taken together with the H4K20ac ChIP-seq data (Fig. 2b,c), our results indicate that genes associated with H4K20ac at TSSs do not exhibit an open chromatin structure.

### H4K20ac is a unique mark

**Figure 3:**
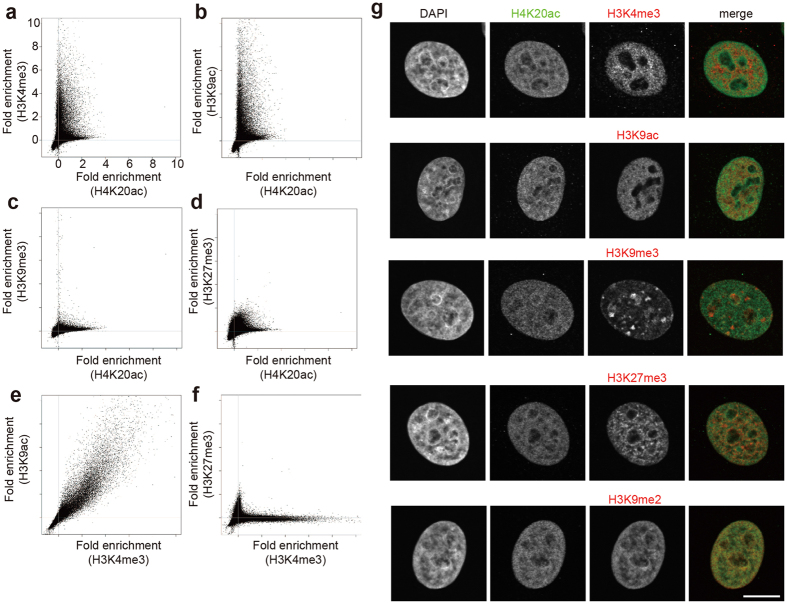
Correlation of H4K20ac with other histone modifications. (**a**–**f**) Correlations of H4K20ac with other histone modifications around the TSS were analyzed and plotted using the ChIP-seq data. (**a**–**d**) see also Supplementary Fig. S3-1). Controls for positive and negative correlations are also shown (**e**,**f**). (**g**) Comparison of H4K20ac distribution with other histone modifications by immunofluorescence confocal microscopy. Scale bar: 10 μm.

As the localization and function of many different histone modifications have been determined, we performed a correlation analysis between H4K20ac and each of the other previously described modifications using the ChIP-seq data. However, none of the tested modifications showed an apparent strong positive correlation with H4K20ac by scatter plot analysis (Fig. 3a–d and Supplementary Fig. S3-1), while H3K4me3 showed positive and negative correlations with H3K9ac and H3K27ac (Fig. 3e,f). To further compare the similarity and diversity between pairs of ChIP-seq signals, we calculated the Jaccard index (Supplementary Fig. S3-2), which represents the size of the intersection divided by the size of the union of the signal-enriched regions of two samples. As expected, H3K27ac showed similarities with active marks, including H3K9ac, H3K4me2, and H3K4me3. In contrast, neither H3K27me3 nor H4K20ac showed much similarity with other modifications. The unique distribution of H4K20ac was also observed at the confocal microscopy level. H4K20ac did not show substantial overlap with most modifications, including those associated with transcriptional activation (H3K4me3 and H3K9ac) or repression (H3K9me3 and H3K27me3) (Fig. 3g). We noticed that H4K20ac showed a partial overlap with H3K9me2, which is involved in euchromatic gene repression^10^, although their patterns were quite different (the former was more punctuate than the latter). This may reflect the broad distribution of H3K9me2 throughout the body of repressed genes and the TSS-enriched distribution of H4K20ac.

### Possible association between H4K20ac and a transcriptional repressor

**Figure 4:**
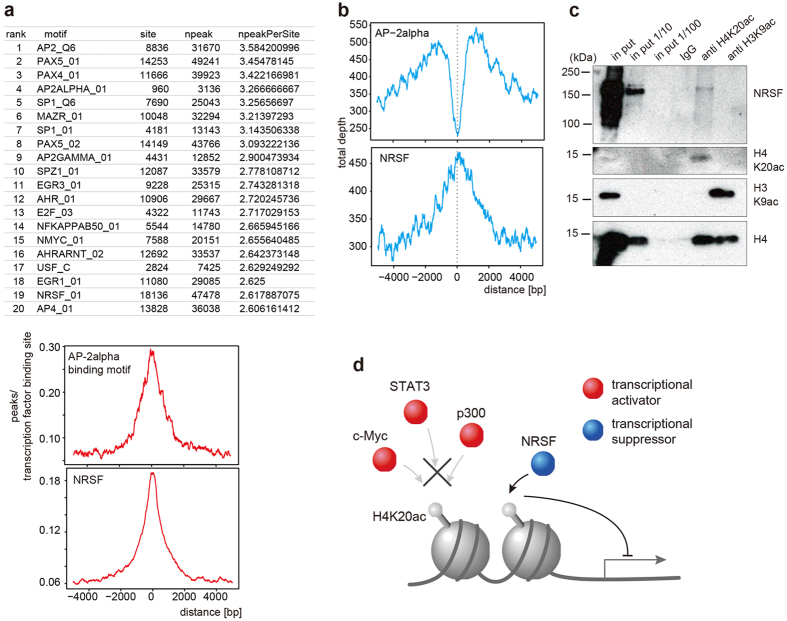
Transcription factor binding sites in H4K20ac-enriched sequences. (**a**) The top 20 binding motifs that were observed in H4K20ac-enriched sequences (upper). University of California Santa Cruz’s Transcription Factor Binding Sites conserved track (hg19, tfbsConsSites) was used for the location definition of cis-elements on the human genome. H4K20ac enrichment across factor binding motif sites for AP2-α (middle) and NRSF/REST (lower) are shown. See Supplementary Fig. S4 for other factors. (**b**) H4K20ac distribution across transcription factor binding sites in HeLa cells by ChIP-seq (AP2-alpha and NRSF). See Supplementary Fig. S4 for other factors. (**c**) ChIP-western. Crosslinked HeLa cell chromatin was immunoprecipitated with control mouse IgG, anti-H4K20ac, and anti-H3K9ac. After de-crosslinking, the input (1×, 1/10×, and 1/100×) and the immunoprecipitates were separated in SDS-polyacrylamide gels and probed with the indicated antibodies. NRSF/REST signal was observed in H4K20ac ChIPed sample. The immunoblotting pictures were cropped from original full length immunoblots (Supplementary Fig. S5). The gels were run under the same experimental conditions. (**d**) Schematic drawing of a possible H4K20ac function in transcriptional repression. Binding of transcriptional activators may be inhibited by H4K20ac.

To gain insights into the mechanistic link between H4K20ac and gene repression, we searched transcription factor binding motifs on H4K20ac-enriched regions using public datasets^11^. As a result, many transcription factor binding motifs were found in the H4K20ac-enriched regions of both HeLa-S3 and hSMC (Fig. 4a and Supplementary Table S1, S2). However, co-localization plots^11^ using the ENCODE ChIP-seq data for HeLa cells^9^ showed that most of them (i.e., AP2-α, AP2-γ, c-Myc, p300, STAT3, c-Jun, Max, ELK1, CEBPB, USF2, E2F4 and NFY) did not coincide with H4K20ac enrichments (Fig. 4b and Supplementary Fig. S4), suggesting that H4K20ac inhibits the binding of such activators and/or their binding prevents H4K20 acetylation. In contrast, a transcriptional repressor, neuron-restrictive silencer factor/repressor element 1-silencing transcription (NRSF/REST), was co-localized with H4K20ac (Fig. 4b), which is consistent with the association of H4K20ac with minimally expressed genes. This apparent difference in co-localization with H4K20ac between NRSF/REST and the other tested transcription factors could be due to the different numbers of false positives that were identified by the motif prediction, but this was unlikely. The higher the ratio of false positives is, the lower the peak becomes, because there is no binning of transcription factors in false positive motifs. However, in cases of AP2-α, AP2-γ, c-myc, and MAX, the positive motifs in the ChIP-seq from HeLa cells were 14.0, 12.5, 3.4, and 6.9% of the predicted motifs, respectively. These coverage rates are higher than, or comparable to, the positive rate for NRSF/REST (4.4%). Thus, the colocalization of H4K20ac with NRSF/REST does not appear to be the result of a different false positive rate than those for the other tested motifs.

To experimentally validate the association of H4K20ac with NRSF/REST, we performed ChIP followed by immunoblotting. NRSF/REST was indeed enriched in the anti-H4K20ac immunoprecipitate but not in the anti-H3K9ac or control IgG immunoprecipitate (Fig. 4c). Therefore, H4K20ac may function as a repressive mark together with transcriptional repressors, such as NRSF/REST (Fig. 4d).

## Discussion

In this study, we discovered that H4K20ac is an unusual acetylation that is enriched in TSSs of minimally expressed genes in human cells. Unlike H3K9me3, a constitutive heterochromatin mark, H4K20ac was less associated with TSSs of completely silenced genes (those with no mRNA expression), suggesting that H4K20ac may play a role in repressing transcribed genes rather than in assisting with heterochromatin formation to induce a fully silent state, possibly preventing methylation on the same residue^12^. Additionally, H4K20ac was also slightly enriched in the body regions of transcribed genes, although the level was much lower than that of H3K36me3, which is thought to be added after RNA polymerase II elongation and to prevent non-promoter initiation. The weak accumulation of H4K20ac in gene body regions may also contribute to repressing non-promoter initiation.

Although the molecular mechanism behind H4K20ac and its involvement in gene repression remains elusive, a transcription repressor NRSF/REST may link H4K20ac and gene repression. Interestingly, NRSF/REST is known as a master regulator of neuronal gene expression in non-neuronal cells^13^. Gene ontology analysis of H4K20ac ChIP-seq data using the DAVID functional annotation tool^14^ also suggested the presence of H4K20ac-enrichment in several signaling pathways in neural cells (calcium signaling, p = 0.000000000601; neuroactive ligand-receptor interaction, p = 0.0000163; axon guidance, p = 0.00667; Supplementary Table S3). Although these possible molecular links between H4K20ac and NRSF/REST are just speculative and need more future study, the discovery of the associations of H4K20ac with NRSF/REST may be important in understanding the function of H4K20ac. It is also required to identify enzymes mediating the acetylation and deacetylation (i.e., acetyl transferase and deacetylase) of H4K20, as well as the reader proteins.

Recent studies have broaden the range of modifications on histone and other proteins^15^,^16^. H3K4, for example, can be acetylated in addition to its well-conserved methylations as active marks for transcription^15^. H3K4ac and H3K4me2/3 are overlapped on transcriptionally active genes, and this is in contrast with the situations of acetylation and methylation with opposed functions on other residues, such as H3K9, H3K27, and H3K36. H3K4ac could activate transcription redundantly with acetylation on other residues, and its spreading is restricted by H3K4me2/3, which may prevent spurious transcriptional initiation^15^. Lysine residues in non-canonical repeat of RNA polymerase II largest subunit are also acetylated and methylated on transcriptionally active genes^17^,^18^. In this case, methylation is observed at the early step of transcription and the conversion from methylation to acetylation may fine tune the transcription regulation. The dynamics balance between acetylation and methylation on H4K20 may also be involved in fine tuning of chromatin regulation at relatively repressed states.

It should be noted that recent discovery of histone acylations, like crotonylation and 2-hydroxyisobutyrylation, on histones have further broadened the complexity of modifications^16^. In post meiotic cells, H4K8 crotonylation at the TSSs appears to be a better mark of active genes than its acetylation^19^. It is interesting to speculate that H4K20 may be acylated in highly active genes while the acetylation is associated with lower expressed genes.

## Methods

### Animals

All institutional and national guidelines for the care and use of laboratory animals were followed. All animal care and experimental procedures in this study were approved by the Hokkaido University Animal Experiment Committee (approval number: 11-0109) and carried out according to guidelines for animal experimentation at Hokkaido University. Animals were housed in a specific pathogen-free facility at Hokkaido University. Humane euthanasia of mice was performed by cervical dislocation by individuals with a demonstrated high degree of technical proficiency.

### Generation, selection, and purification of monoclonal antibodies against H4K20ac

A synthetic peptide (GKGGAKRHR(ac-K)VLRDNIQGIC; Sigma-Genosys, Ishikari, Japan) was conjugated to keyhole limpet hemocyanin and employed to immunize mice^20^. After producing hybridomas, clones were screened by ELISA using plates coated with the synthetic peptide (acetylated or unacetylated) conjugated with bovine serum albumin (BSA). After re-cloning, supernatants from clones whose antibodies reacted with the specifically-modified peptide were used to probe blots prepared using the total protein from HeLa cells lysed in SDS-gel loading buffer^21^. The supernatants that gave a single band at the expected size of histone H4 were selected for immunofluorescence examination to see if they were able to stain the nucleus. Clones passing through these successive screens were further analyzed by ELISA using various H4 peptides^22^, with serial dilutions against the BSA-conjugated peptides. The clone with the highest specificity to the acetylated peptide was selected (clone CMA424). The isotype of this antibody was determined using a kit according to the manufacturer’s instructions (MMT1, Serotec, Raleiph, NC, USA). Hybridoma cells were routinely maintained in GIT medium (Wako Pure Chemical Industries, Ltd, Osaka, Japan). For antibody purification, cells were grown in CD Hybridoma medium (Thermo Fisher Scientific, Waltham, MA, USA) and the supernatant (250 ml) was passed through a 0.45 μm-pore filter. The filtrated supernatant was directly poured into a HiTrap Protein A HP Sepharose column (1 ml; GE Healthcare, Little Chalfont, UK). After washing with PBS, IgG was eluted using IgG Elution buffer (Thermo Fisher Scientific), and the pH of the buffer was immediately made neutral with Tris. IgG was concentrated up to approximately 2 mg/ml in PBS with an Amicon Ultra filter (50 k cut-off; EMD Millipore, Billerica, MA, USA).

### Other antibodies

Mouse monoclonal antibodies directed against H3K4me3, H3K9me3, H4K9ac, H3K9me2, H3K9ac that have been described in previous papers^23^,^24^ were also used in this study. A rabbit monoclonal anti-NRSF/REST antibody (EPR2436Y) was purchased from Abcam (Cambridge, UK).

### Cells

Human Hela-S3 (JCRB9010) and aortic smooth muscle cell line (hSMC) were purchased from the National Institute of Biomedical Innovation, Japan, and Lonza (Basel, Switzerland), respectively. Hela-S3 cells were maintained according to the protocol from the ENCODE group (http://genome.ucsc.edu/encode/protocols/cell/human/HeLa-S3_IFN_Crawford_protocol.pdf). To prepare interferon γ-treated cells, cells were incubated in 5 ng/ml interferon γ for 4 hours. hSMCs were maintained with DMEM (Thermo Fisher Scientific) supplemented with 10% FCS (Thermo Fisher Scientific).

### Separation of a histone H4 K20-containing peptide for mass spectrometry

Histone H4-H3 complex was prepared from HeLa-S3 cells grown on 25 150 mm dishes using a Histone Isolation Kit according to the manufacturer’s instruction (Active Motif, Carlsbad, CA, USA). Chromatographic separations were performed using an Agilent 1100 series system (Agilent Technologies, Santa Clara, CA, USA) at 40 °C. UV absorbance was monitored at 214 and 280 nm. For isolation of histone H4, the H3-H4 sample (20 μl) was applied to a Unison CD-C18 column (1 mm i.d. × 150 mm, Imtakt Co., Kyoto, Japan), which had been equilibrated with 5% acetonitrile in 0.1% trifluoroacetic acid. H4 was eluted using a linear gradient of acetonitrile (5–33% in 40 min) at a flow rate of 70 μL/min. Eluted fractions were collected every minute using a fraction collector (FC204, Gilson Inc, Middleton, WI, USA). The fractions containing H4 were identified by western blotting and a MALDI-TOF/TOF (4700 proteomics analyzer, Applied Biosystems, Framingham, MA). Approximately 10 nmol of histone H4 in 50 mM (NH_4_)_2_CO_3_ was digested by 50 pmol of ASP-N (Roche, Basel, Switzerland) for 18 h followed by treatment with 50 pmol of Lysyl Endopeptidase (Wako) for 18 h, which eventually gave the target peptide (RHR^20^KVLR). The digest was separated in the same system, but with a Cadenza CD-C18 column (1 mm × 150 mm, Imtakt Co., Kyoto, Japan) at a flow rate of 90 μl/min. For identification of the type of modification in the target peptide, synthetic peptides with the same sequence but with distinct modifications (dimethylation, trimethylation, and acetylation) at K20 were prepared and separated under the same conditions.

### Matrix-assisted laser desorption/ionization (MALDI) mass spectrometry

The peptides were applied onto the flat surface of a stainless steel MALDI sample plate. Thereafter, the matrix solution (5 mg/mL of a-CHCA) was blotted. The accurate mass measurements were carried out in positive-ion mode with a MALDI-TOF/TOF (4700 proteomics analyzer, Applied Biosystems). Ions were generated by irradiating the sample area with a 200 Hz Nd:YAG laser operated at 355 nm. The internal calibrants were as follows: bradykinin (MH^+^: 1060.5687), RHR^20^ (dimethyl-K)VLR (histone H4, 17–23; MH^+^: 992.6588), and PHRYRPGTVA (histone H3, 38–47; MH^+^: 1153.6225).

### Native chromatin immunoprecipitation (Native ChIP) and ChIP-seq

Nucleosomes were prepared as previously reported^23^. Briefly, hSMC cells (2 × 10^7^ cells) and spinner-adapted Hela-S3 cells (2.5 × 10^7^ cells), grown on four 225 cm^2^ dishes, were collected (360 × g; 5 min; 4 °C), washed with ice-cold RSB (10 mM HEPES-NaOH [pH 7.4], 15 mM NaCl, 1.5 mM MgCl_2_), centrifuged (640 × g; 10 min; 4 °C), and resuspended in 10 ml ice-cold RSB containing 1% Triton X-100 and a protease inhibitor cocktail (Nacalai Tesque, Kyoto, Japan). Nuclei were prepared by homogenization using a Dounce homogenizer (tight pestle; 5 times) and collected by centrifugation (1,700 × g; 15 min; 4 °C). After washing twice with 5 ml buffer A (15 mM HEPES-NaOH [pH 7.4], 15 mM NaCl, 60 mM KCl, 0.34 M sucrose, 0.5 mM spermine, 0.15 mM spermidine, 1 mM dithiothreitol, protease inhibitor cocktail; Nacalai Tesque), nuclei were resuspended in 1 ml of buffer A and digested with 0.5 μl of micrococcal nuclease (NEB, Ipswich, MA, USA) in the presence of 1 mM CaCl_2_ and 1 μM Tricostatin A (Sigma-Aldrich, St.Louis, MO, USA) at 30 °C for 2 h (agitating every 20 min). After terminating the nuclease reaction by adding 20 μl of 0.5 M EDTA [pH 8.0] and centrifugation (10,000 × g; 10 min; 4 °C), the pellet was suspended in 500 μl of 10 mM EDTA [pH 8.0] and 0.5 M NaCl. After centrifugation (20,000 × g; 5 min; 4 °C), the supernatants containing nucleosomes were collected and used for ChIP.

Control mouse IgG (Sigma-Aldrich) or anti-H4K20ac antibody (40 μg) was incubated with 180 μl of an original suspension of Dynabeads M-280 Sheep anti-Mouse IgG (Thermo Fisher Scientific) in 1 ml of RIPA-150 buffer (50 mM Tris-HCl [pH 8.0], 150 mM NaCl, 1 mM EDTA, 0.1% SDS, 1% Triton X-100, 0.1% sodium deoxycholate) at 4 °C for 4 h. For ChIP, 250 μl of the nucleosome fraction was mixed with 1 ml buffer B (20 mM Tris-HCl [pH 8.0], 5 mM EDTA, 500 mM NaCl, and 0.2% Tween 20) and incubated with the antibody-bound beads. After overnight incubation at 4 °C with rotation, the beads were washed once with 1 ml of ice-cold RIPA-150, once with 1 ml of RIPA-500 (containing 500 mM NaCl), and twice with 1 ml of TE (10 mM Tris-HCl [pH 8.0] and 1 mM EDTA). The washed beads were incubated in 200 μl of ChIP elution buffer (10 mM Tris-HCl [pH 8.0], 300 mM NaCl, 5 mM EDTA [pH 8.0], 0.5% SDS) at 65 °C for 8 h. After RNase A treatment (5 μg/ml; 30 min; 37 °C) followed by proteinase K treatment (0.2 mg/m; 2 h; 55 °C), DNA was extracted using phenol/chloroform and recovered by ethanol precipitation. Sequencing was performed as previously described^25^. ChIP-seq and mRNA-seq data were submitted to DDBJ Sequence Read Archive with the accession number: DRA004456. The statistics and quality check results of ChIP-seq and mRNA-seq were summarized in Supplementary Table S4.

### Cross-linked chromatin immunoprecipitation (ChIP) and western blotting

Cross-linked ChIP was performed as previously described^23^. HeLa-S3 cells grown on twenty 150 mm dishes were cross-linked with 1% formaldehyde (Electron Microscopy Sciences, Hatfield, PA, USA) in medium (10 ml per dish) for 5 min at room temperature, and then incubated in 200 mM glycine in medium (10 ml per dish) for 5 min to quench any reactive aldehydes. Cells were rinsed with PBS, immersed in lysis buffer (10 mM Tris-HCl [pH 8.0], 10 mM NaCl, and 0.5% NP-40; 10 ml per dish) for 10 min, and harvested using a cell scraper with lysis buffer (0.5 ml per dish). Cells were collected by centrifugation (1,000 × g; 3 min; 4 °C) and resuspended in 50 μl SDS lysis buffer (50 mM Tris-HCl [pH 8.0], 10 mM EDTA, and 1% SDS). After mild rotation for 10 min at 4 °C, 450 μl ChIP dilution buffer (50 mM Tris-HCl [pH 8.0], 167 mM NaCl, 1.1% Triton X-100, 0.1% sodium deoxycholate, and protease inhibitor cocktail [complete EDTA-free; Roche]) was added before sonication (Sonifier SLPe with microtip, Branson, Danbury, CT, USA; 8 times for 55 sec each; Amplitude 16%). After centrifugation (20,000 × g; 20 min; 4 °C), 21 ml RIPA-150 was added to a total of 10 ml of supernatant to yield the input for ChIP. After taking an aliquot (1 ml) for the input sample, the rest was divided into 3 parts (10 ml per ChIP reaction).

For cross-linked ChIP, Dynabeads M-280 sheep anti-mouse IgG (Thermo Fisher Scientific; 500 μl original suspension) were incubated with 10 μg anti-H4K20ac, anti-H3K9ac, or normal mouse IgG (Jackson Immunoresearch, West Grove, PA, USA) in 500 μl RIPA-150 for 3 h at 4 °C with rotation and washed twice with 1 ml RIPA-150. ChIP input (10 ml) was incubated with IgG-bound Dynabeads overnight at 4 °C with rotation. Beads were washed 3 times with 10 ml RIPA-150, mixed with 200 μl 2 × SDS-gel loading buffer, and incubated overnight at 65 °C to reverse cross-linking. After boiling for 10 min, the supernatant was separated in 4–20% gradient or 7% SDS-polyacrylamide gels, and transferred to PVDF membranes (Pall, Port Washington, NY, USA or GE Healthcare). Membranes were washed 3 times in TBST (20 mM Tris-HCl [pH 8.0], 150 mM NaCl, and 0.05% Tween 20) for 5 min, blocked for 30 min in Blocking-One (Nacalai Tesque; 30 min), and washed 3 times for 5 min each in TBST. Membranes were incubated for 1 hr at room temperature with anti-NRSF/REST (1:500) and anti-histone antibodies (1 μg/ml) in Can-get-signal (Toyobo, Osaka, Japan), washed with TBST (3 times for 10 min each), incubated with peroxidase-conjugated anti-rabbit IgG or anti-mouse IgG (1:5,000; Dako, Troy, Michigan, USA) in Can-get-signal (1 h), and washed with TBST (3 times for 10 min each). Signals were developed using ECL plus reagent (Pierce) and detected using Hyperfilm ECL (GE Healthcare).

### Immunofluorescence and microscopy

HeLa cells grown on a cover slip were fixed with 4% formaldehyde (Electron Microscopy Sciences) in 250 mM HEPES-NaOH (pH 7.4; Wako) containing 0.1% Triton X-100 for 10 min at room temperature. Fixed cells were permeabilized with 1% Triton X-100 in PBS for 20 min at room temperature and washed three times with PBS. After blocking with 10% Blocking One-P (Nacalai Tesque) in PBS for 20 min at room temperature, cells were incubated in 2 μg/ml dye-conjugated antibodies and 100 ng/ml Hoechst33342 in PBS for 2 h at room temperature, and washed three times with PBS. All fluorescence images were collected using a confocal microscope (FV1000; Olympus, Tokyo, Japan) operated by the built-in FV1000 software (ver.3.1a). Fluorescence images of Cy5, Cy3, Alexa Flour 488, and Hoechst33342 were sequentially collected with a 60× PlanApoN (NA 1.40) oil-immersion lens (8× zoom; 512× 512 pixels; 12.5 μs/pixel; 4 line Kalman; 12-bit; pinhole 120 μm).

### ChIP-seq data analysis

Sequenced reads were mapped onto the human genome (hg19) using Bowtie (version 0.12.8; parameters of ‘–v 3 –m 1′); we used unique mapped reads and allowed mismatches of up to 3 bases. The significantly enriched regions of the H4K20ac signals (peaks) were identified using MACS (version 1.4.1; default parameters). To calculate ChIP-seq signal intensity (*SI*), we counted mapped reads every *L* bp intervals (bins) on the genome, and expressed the read count numbers as RPKM (Reads Per Kilobase Per Million reads) regardless of the bin size. The RPKM difference between ChIP and input-DNA control data (RPKM_ChIP_ − RPKM_Input_) was used as the normalized ChIP-seq signal intensity. We also used a histone modification ChIP-seq dataset of HeLa-S3 cells provided by ENCODE/Broad institute (GEO: GSE29611)^26^.

ChIP-seq and FAIRE-seq data were visualized with the peak distribution plot of gene loci (Fig. 2b–d, and Supplementary Fig. S2-1d, S2-2b,d, S2-5b,c). The “peaks per gene” (*y*-axis) is the group-average number of piled-up peak at the relative base pair position from TSS or transcription end site (TES). The peaks were called by MACS with default parameters. The data used for peak calling were the HeLa-S3 H4K20ac ChIP-seq data (Fig. 2b,c), HeLa-S3 FAIRE-seq data (Fig. 2d), and hSMC H4K20ac data (supplied as treatment ‘–t’ in MACS parameter) (Supplementary Fig. S2-5b,c) using the respective input control data for each (supplied as control ‘–c’). All annotated human genes were categorized into eleven groups based on their expression levels in HeLa-S3 or hSMCs, as described below in “mRNA-seq data analysis”. The plots for whole gene ±5 kbp (Fig. 2c, and Supplementary Fig. S2-1c,d, S2-3b, S2-5c) were drawn by using the relative distance between TSS to TES for the regions within gene bodies and the actual distance of ±5 kbp for the regions outside of the genes.

For Fig. S2-1d, the “peaks per gene” (*y*-axis) was calculated as the group-average of peak-numbers called by MACS utilizing the HeLa-S3 H4K20ac data. The gene groups separated by deciles of H3K4me3 signal level were determined using the *SI*s (RPKM difference in a window) of ENCODE/Broad H3K4me3 (Rep1) data on all TSS ±2 kbp regions. ENCODE/Broad HeLa-S3 control data for the Rep1 ChIP-seq was used for normalization. The *SI*s of the data were calculated as the RPKM difference, described above. In Supplementary Fig. S2-2 and S2-3b, the “broadPeak” data of ENCODE/Broad HeLa-S3 H3K4me3, H3K9me3, and H3K36me3 were obtained from UCSC (http://hgdownload.cse.ucsc.edu/goldenPath/hg19/encodeDCC/wgEncodeBroadHistone/) and used for plots.

For Supplementary Fig. S2–3a, a boxplot of fold-changes (FC) for the eleven gene expression groups was generated. H4K20ac ChIP-seq and its Input data were utilized for calculating the RPKM in the selected regions of the genome, which are “TSS ±1 kbp” and “gene-body (1 kb downstream of TSS to TES)”. Then, the FC was calculated as RPKM_ChIP_/RPKM_Input_ on each selected region and expressed in box plots.

Aggregation plots were also drawn using the raw signal distribution (not MACS peaks) of H4K20ac (Supplementary Fig. S2-1b,c). The *SI*s of H4K20ac were calculated in each 200 bp window (~1 nucleosome plus linker size) of the whole genome. Then, the *SI* for every window was piled-up around TSSs for the 11 gene groups that were categorized based on their mRNA levels.

### Consistency measurement using ENCODE/Broad Institute histone modification data set

We first set true (*SI* ≥threshold) or false (*SI* <threshold) labels on each 10 kb window on the genome as “matching control” labels. The threshold was fixed to the 95th percentile of *SI*s in all windows when the control was ENCODE H3K4me3 and H3K27ac, because these signals show spiky distributions. The threshold was fixed to the 90th percentile of *SI*s for ENCODE H3K9me3, H3K27me3, and HeLa-S3 H4K20ac data, because these signals distribute more broadly and dispersedly. Similarly, we set positive (*SI* ≥cutoff) or negative (*SI* <cutoff) labels in all windows with sequential settings of cutoff levels within the range of *SI* ∈ [−1, 2]. In each level of the cutoff, we counted the number of true-positive (TP) and false-positive (FP) by matching the labels on windows of all histone modification data sets (ENCODE and our H4K20ac) against the labels of matching controls. The Jaccard index, *J* ∈ [0, 1], measures the similarity between two sets, *A* and *B*, calculated as #(*A* ∩ *B*)/#(*A* ∪ *B*). If the sets *A* and *B* were regarded as true and positive labels respectively, *J* is also calculated as #*TP/*(#*true*+*#FP*) our analysis. For example, *J* = *1* if all the positive labels completely match all the true labels of a matching control (*A* = *B*), and *J* = *0* if all the positive labels were on the false labels (#(*A* ∩ *B*) = 0. We plotted the curves of Jaccard index as a function of the cutoff levels.

### mRNA-seq data analysis

The mRNA-seq library was prepared by using TruSeq Stranded mRNA Sample Prep Kits (Illumina, San Diego, CA, USA) following the manufacturer’s protocol. Sequenced reads were mapped with Tophat software (version 1.4.1), and analyzed using Cufflinks (version 1.3.0). We defined expression groups using the fragments per kilobase per million fragments mapped (FPKM) values of genes as estimated by Cufflinks^27^,^28^. A group of genes with FPKM = 0 was labeled as “zero”; the others (FPKM >0 genes) were separated into ten groups by decile intervals based on their FPKM value (q0–10%, q10–20%, …, q90–100%). The columns in Table S4 were extracted from a gene_exp.diff table produced by Cufflinks.

## Additional Information

**How to cite this article**: Kaimori, J.-Y. *et al*. Histone H4 lysine 20 acetylation is associated with gene repression in human cells. *Sci. Rep.* **6**, 24318; doi: 10.1038/srep24318 (2016).

## Supplementary Material

Supplementary Figures

Supplementary Tables
