# Supplementary material for: Histone H4 lysine 20 acetylation is associated with gene repression in human cells
Source: Sci Rep. 2016 Apr 11;6:24318. doi: 10.1038/srep24318 (PMC4827026; doi:10.1038/srep24318)
Supplement: Supplementary Figures [file srep24318-s1.pdf]

Supplementary Figures of:

Histone H4 lysine 20 acetylation is associated with gene repression in human cells.

Jun-Ya Kaimori [1,2,+,*], Kazumitsu Maehara [3,+], Yoko Hayashi-Takanaka [4,5,6], Akihito Harada [3], Masafumi Fukuda [7], Satoko Yamamoto [2], Naotsugu Ichimaru [1], Takashi Umehara [8], Shigeyuki Yokoyama [9], Ryo Matsuda [10], Tsuyoshi Ikura [10], Koji Nagao [11], Chikashi Obuse [11], Naohito Nozaki [12], Shiro Takahara [1], Toshifumi Takao [7], Yasuyuki Ohkawa [3, 6], Hiroshi Kimura [4,5,6,*], and Yoshitaka Isaka [2]

[1] Department of Advanced Technology of Transplantation and [2] Department of Nephrology, Osaka University Graduate School of Medicine, 2-2 Yamadaoka, Suita, Osaka 565-0871, Japan
[3] Division of Transcriptomics, Medical Institute of Bioregulation, Kyushu University, Fukuoka 812-8582, Japan
[4] Graduate School of Frontier Biosciences, Osaka University, 1-3 Yamadaoka, Suita, Osaka 565-0871, Japan
[5] Graduate School of Bioscience and Biotechnology, Tokyo Institute of Technology, 4259 Nagatsuta-cho, Midori-ku, Yokohama 226-8501, Japan
[6] CREST, JST, 4-1-8, Honcho, Kawaguchi, Saitama 332-0012, Japan
[7] Institute for Protein Research, Osaka University, Yamadaoka 3-2, Suita, Osaka 565-0871, Japan
[8] RIKEN Center for Life Science Technologies and [9] RIKEN Structural Biology Laboratory, 1-7-22 Suehiro-cho, Tsurumi-ku, Yokohama, Kanagawa 230-0045, Japan
[10] Radiation Biology Center, Kyoto University, Yoshidakonoe-cho, Sakyo-ku, Kyoto 606-8501, Japan
[11] Graduate School of Life Science, Hokkaido University, Kita 21 Nishi 12, Sapporo 001-0021, Japan
[12] MAB Institute Inc., Kita 21 Nishi 12, Sapporo 001-0021, Japan

+ these authors contribute equally to this work

* corresponding authors
Jun-ya Kaimori M.D., PhD. Tel: +81-6-6879-3746; Fax: +81-6-6879-3749;
E-mail: kaimori@att.med.osaka-u.ac.jp
Hiroshi Kimura, PhD. Tel: +81-45-524-5742; E-mail: hkimura@bio.titech.ac.jp

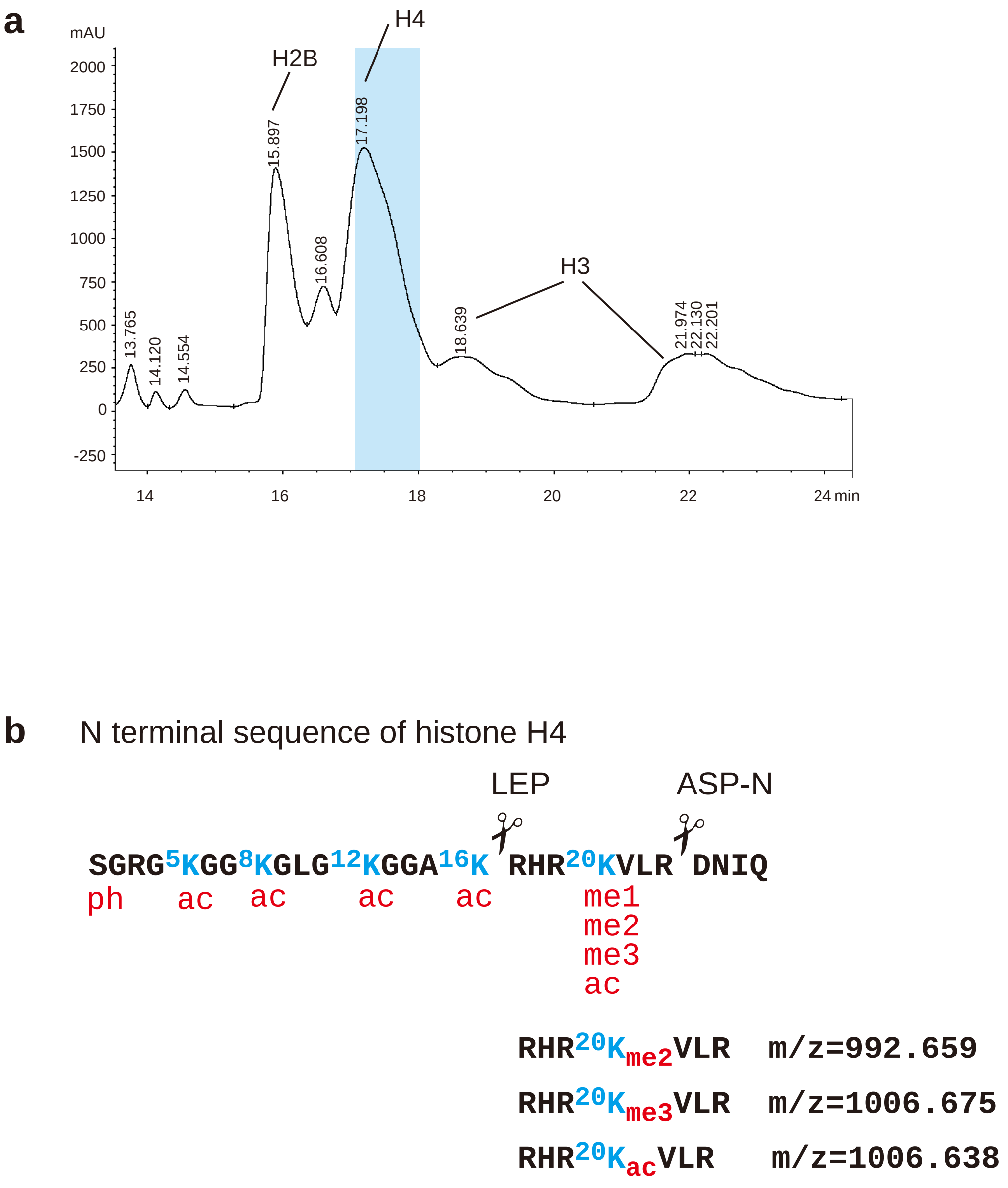

Supplementary Figure S1. Sample preparation for mass spectrometry.
a, HPLC profile of isolated H4-H3 complex. Each solid bar denotes a collected fraction. b, The N-terminal sequence of histone H4 and the peptides expected by the enzymatic digestions. me2: dimethyl; me3: trimethyl; ac: acetyl; LEP: lysyl endopeptidase; ASP-N: endoproteinase Asp-N.

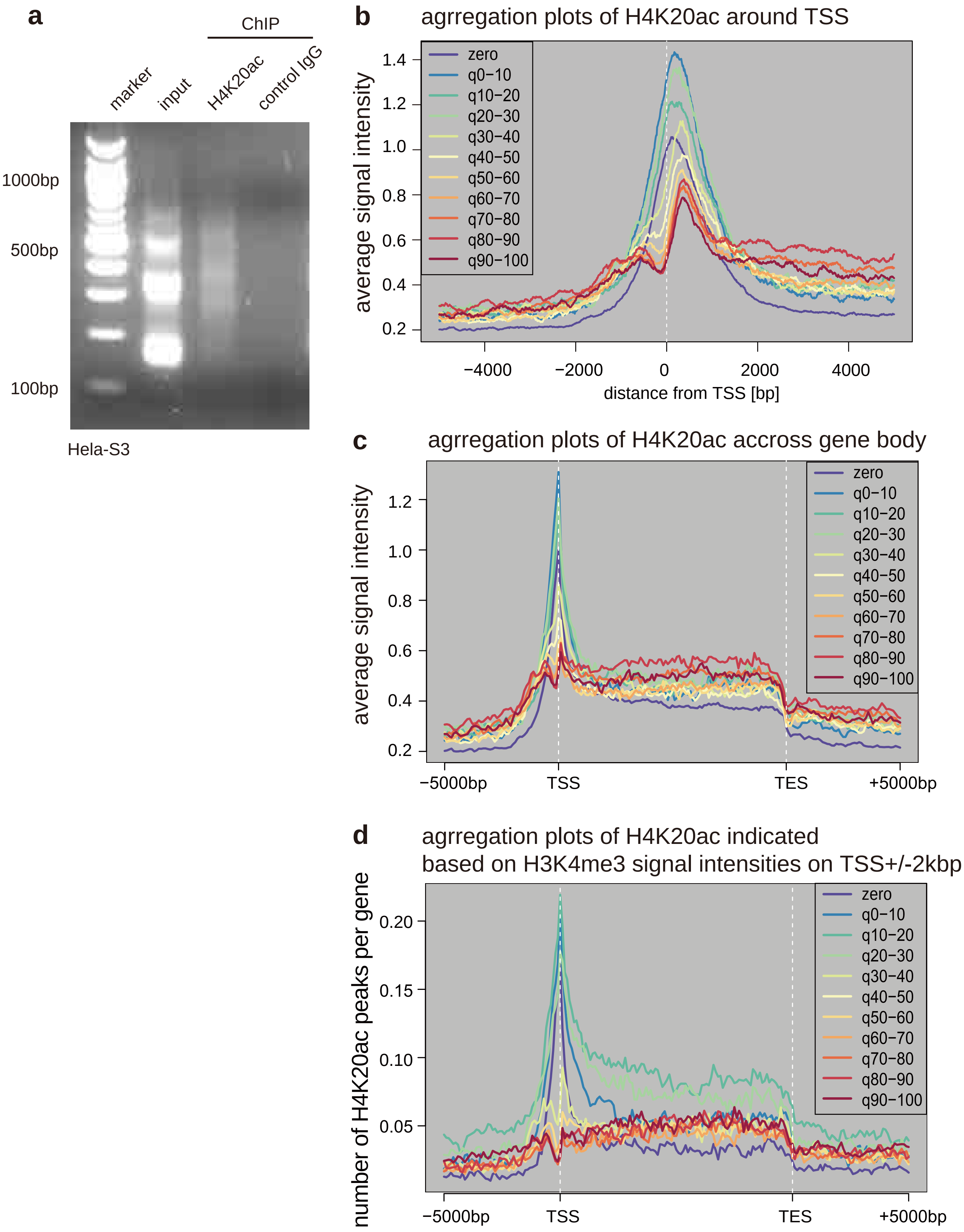

Supplementary Figure S2-1. ChIP-seq analysis: gel electrophoresis and alternative aggregation plot analysis. Native ChIP using Hela-S3 cells maintained in a spinner culture flask. a, 2.0% agarose gel electrophoresis of DNA from input and ChIPed materials with anti-H4K20ac and mouse control IgG. b and c, Averaged signal intensity of H4K20ac signals surrounding the transcription start site (TSS; b) and across the gene bodies (c) were indicated for 11 groups of genes divided based on their expression levels. d, Averaged peak counts of H4K20ac signals across the gene bodies are indicated for 11 group of genes that were based on their H3K4me3 ChIP-seq signal intensity in Hela S3 cells.

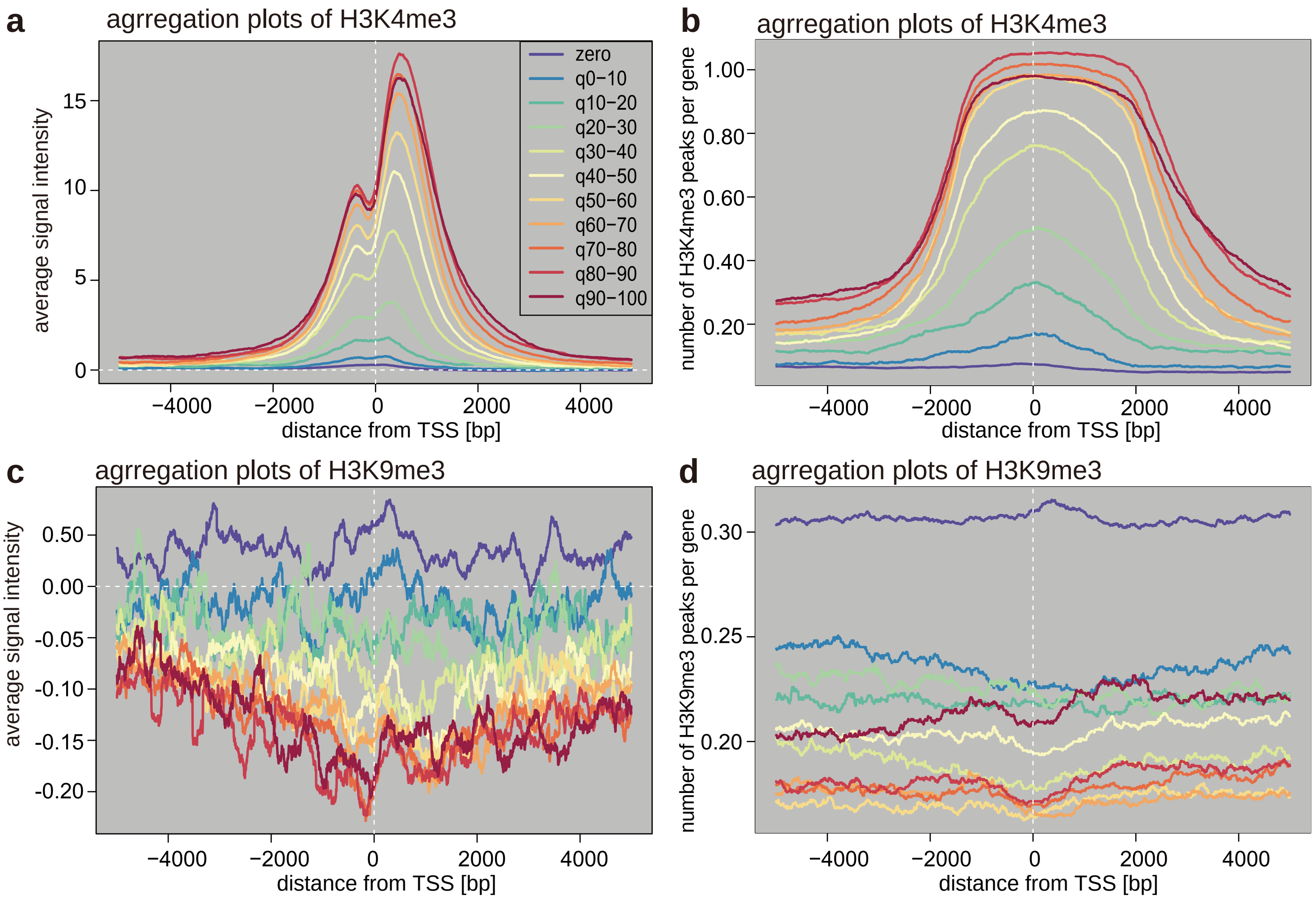

Supplementary Figure S2-2. ChIP-seq analysis: controls.
a–d, Average signal intensity of H3K4me3 (a) or H3K9me3 (c) ChIP-seq signals and averaged peak counts of H3K4me3 (b) or H3K9me3 (d) ChIP-seq signals around TSS were indicated for 11 groups of genes that were divided based on their gene expression in Hela S3 cells.

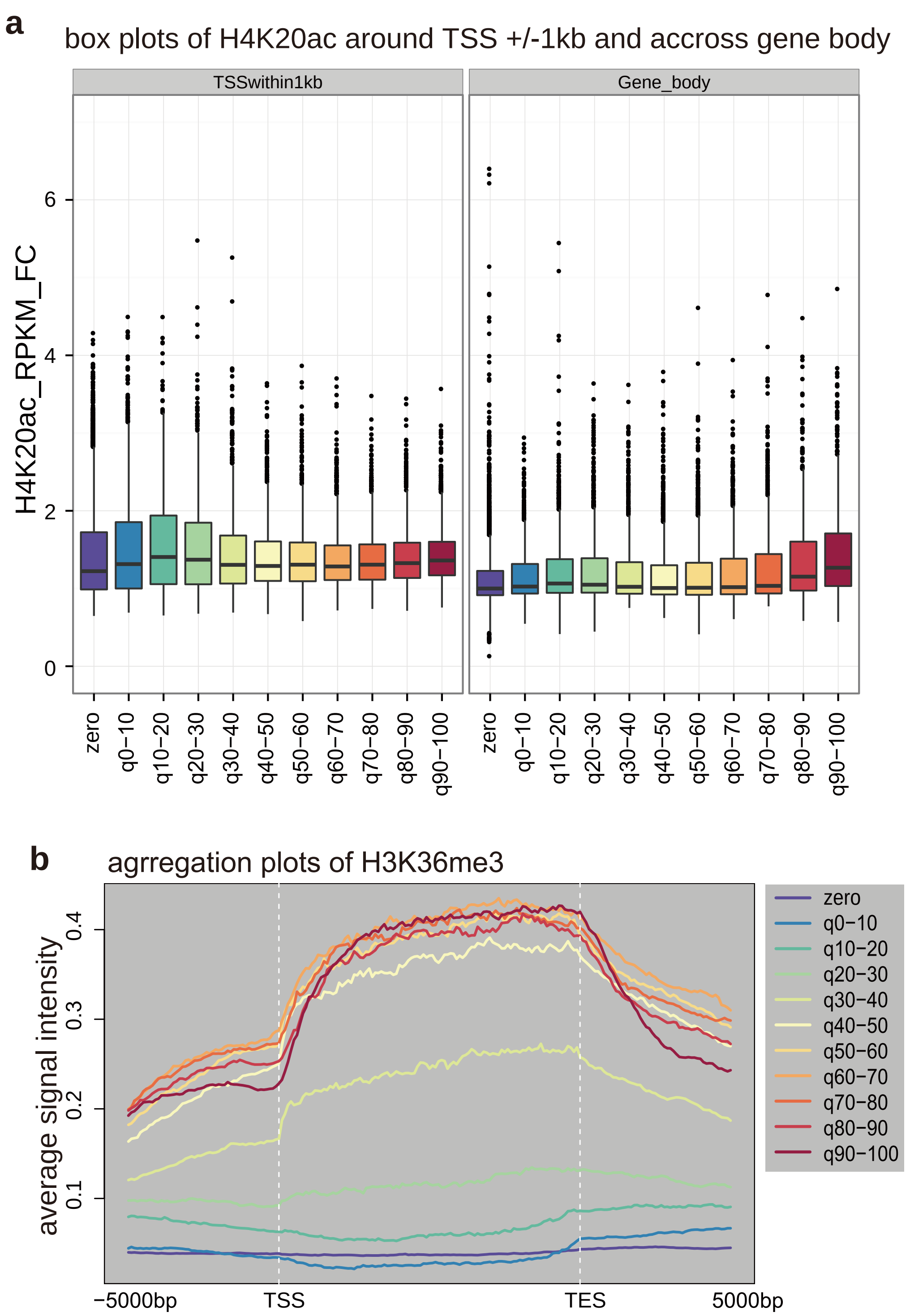

Supplementary Figure S2-3. ChIP-seq analysis: box plot analysis and aggregation plots for H3K36me3. a, H4K20ac signal fold change (ratio of ChIP RPKM to Input RPKM) within 1 kb from TSS (left) and gene body (from 1 kb downstream of TSSs to TESs; right) was calculated for each gene. The distributions in 11 groups of genes, that are categorized based on their gene expression levels, are box-plotted. b, Averaged peak counts of H3K36me3 ChIP signals across the gene bodies are indicated for the same 11 groups of genes described in (a).

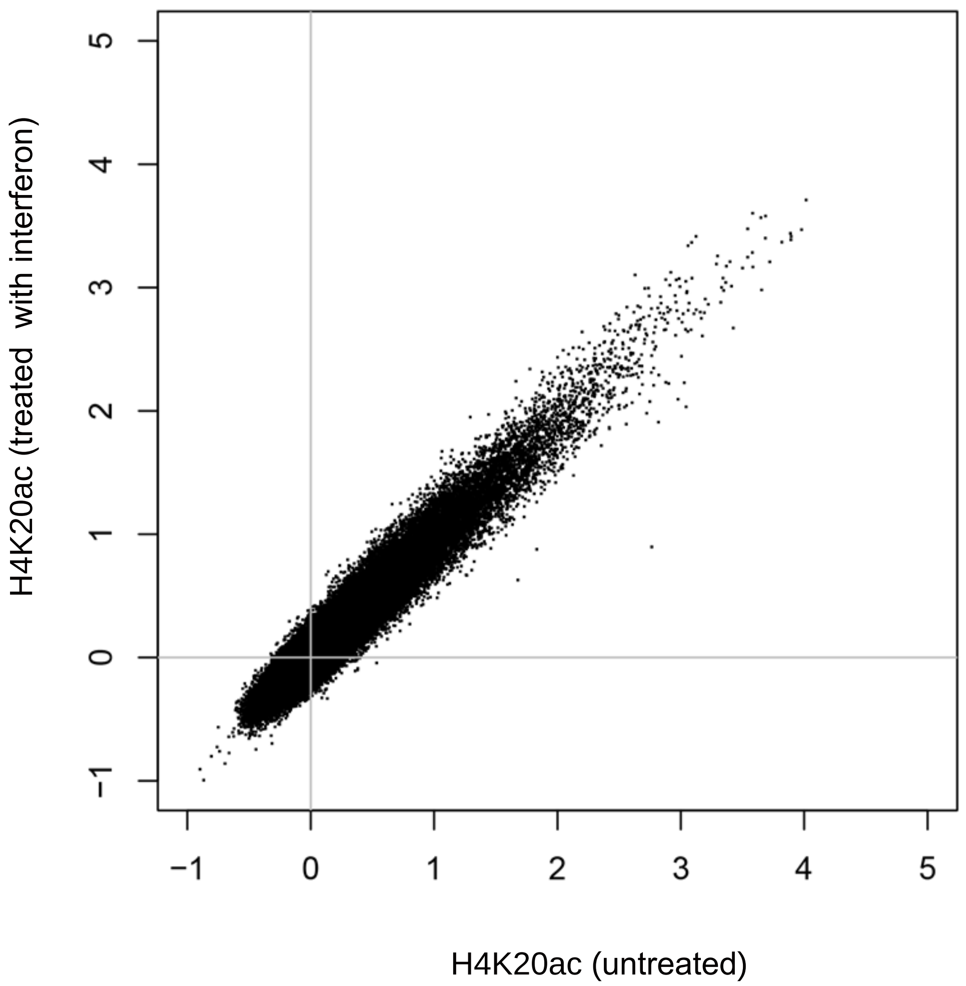

Supplementary Figure S2-4. A scatter plot of H4K20ac in Hela-S3 cells untreated and treated with interferon γ. Genome wide H4K20ac ChIP-seq signal intensities were compared in 10 kb bins between Hela-S3 cells untreated (x-axis) and treated (y-axis) with interferon γ.

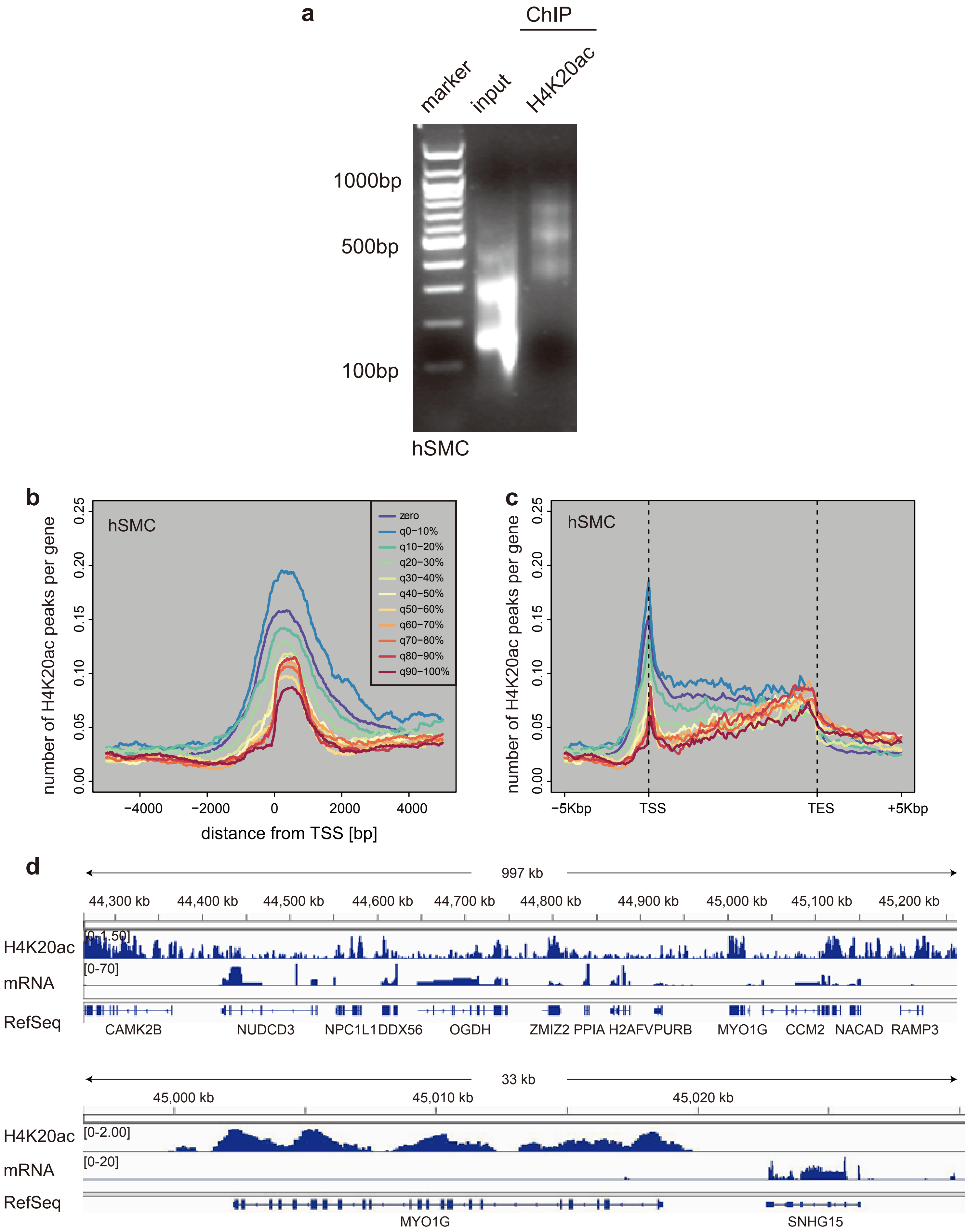

Supplementary Figure S2-5. ChIP-seq analysis: hSMC.
a, 2.0% agarose gel electrophoresis of input and ChIPed DNA with anti-H4K20ac antibody using hSMC. b–c, Averaged peak counts of H4K20ac signals surrounding the TSS (b) and across the gene bodies (c) were indicated for 11 groups of genes that were divided based on their gene expression in hSMC cells. d, Distribution of H4K20ac and mRNA at genome loci spanning 997 kb (upper panel) and 33 kb (lower panel) in hSMC cells.

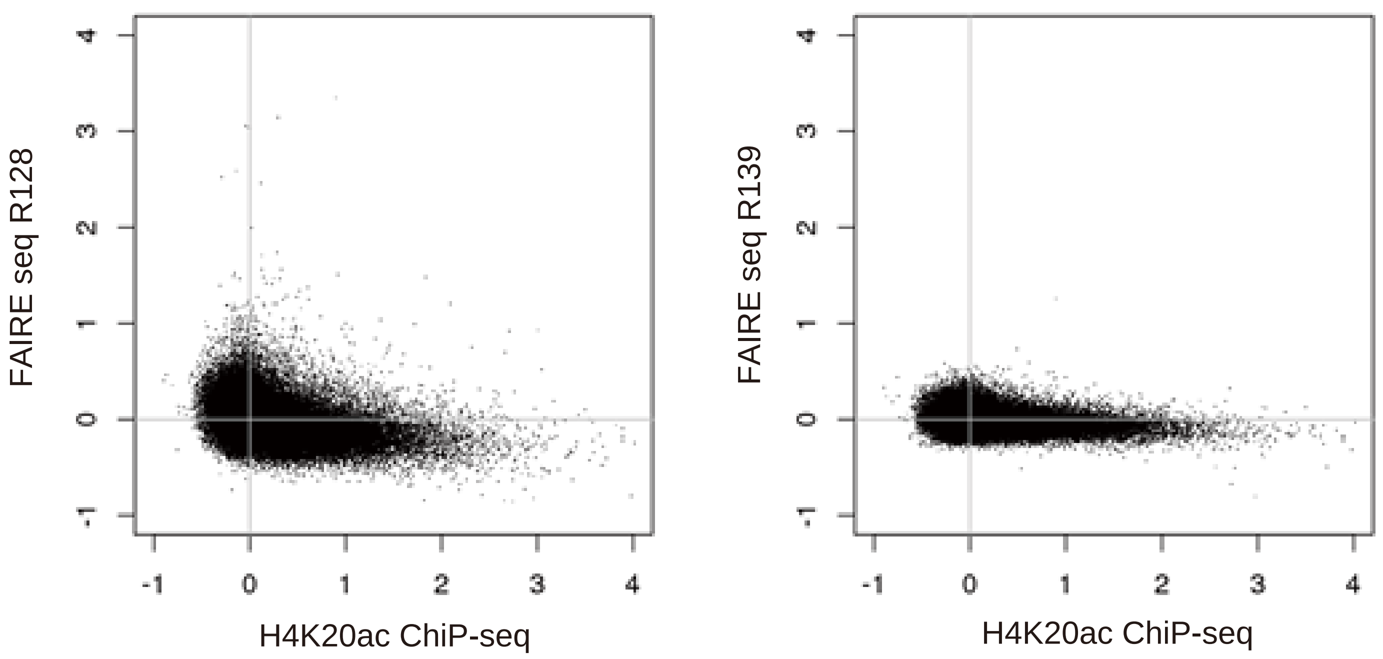

Supplementary Figure S2-6. ChIP-seq: comparison with FAIRE-seq.
Correlation of H4K20ac with FAIRE-seq in the genome wide area. R128, R139: data number of FAIRE-seq of Hela-S3 cells in ENCODE.

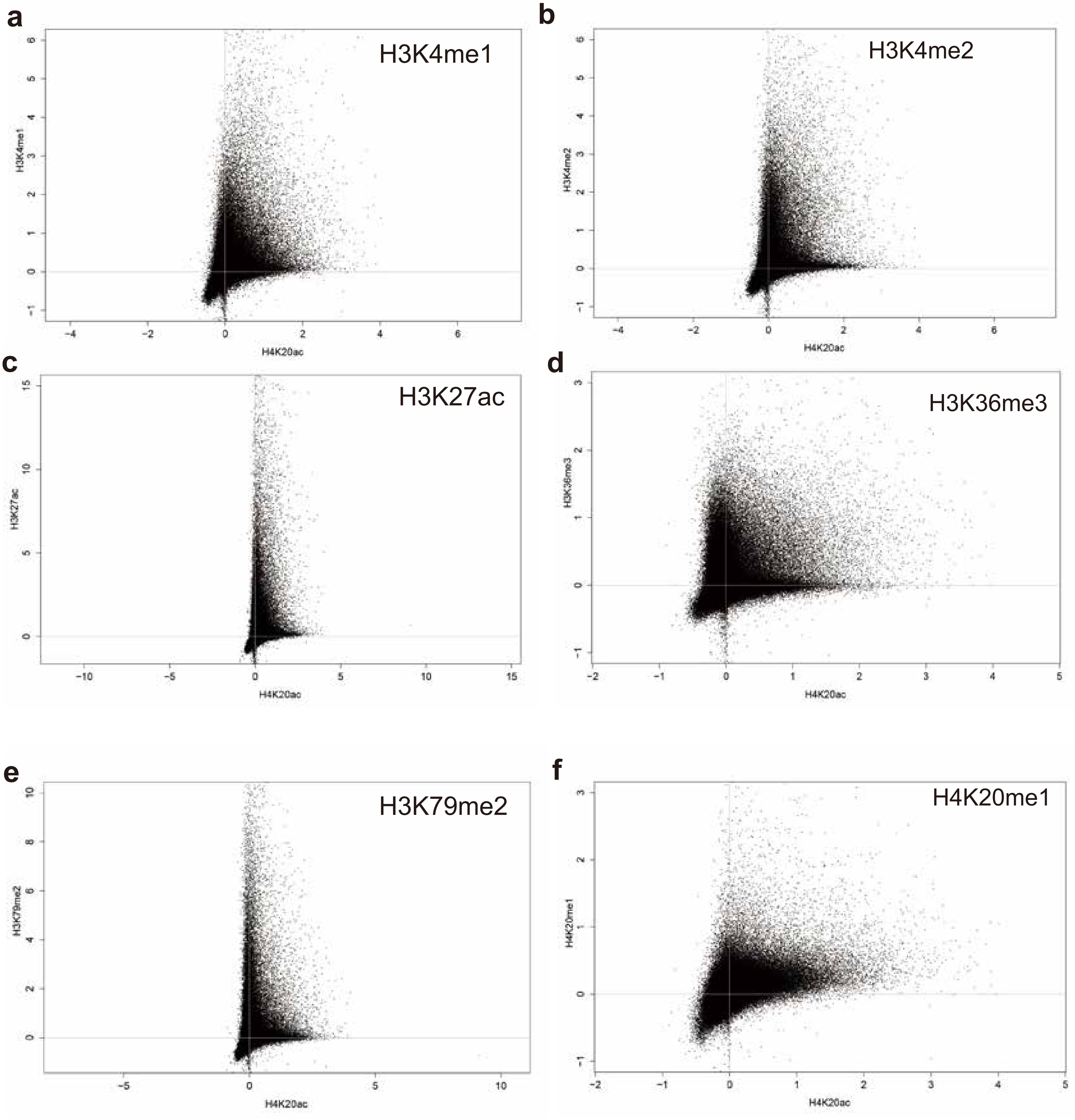

Supplementary Figure. S3-1. Colocalization analysis: scatter plots.
a–f, Scatter plots of H4K20ac with other histone modifications (H3K4me1 (a), H3K4me2 (b), H3K27ac (c), H3K36me3 (d), H3K79me2 (e), H4K20me1 (f)) in the promoter areas.

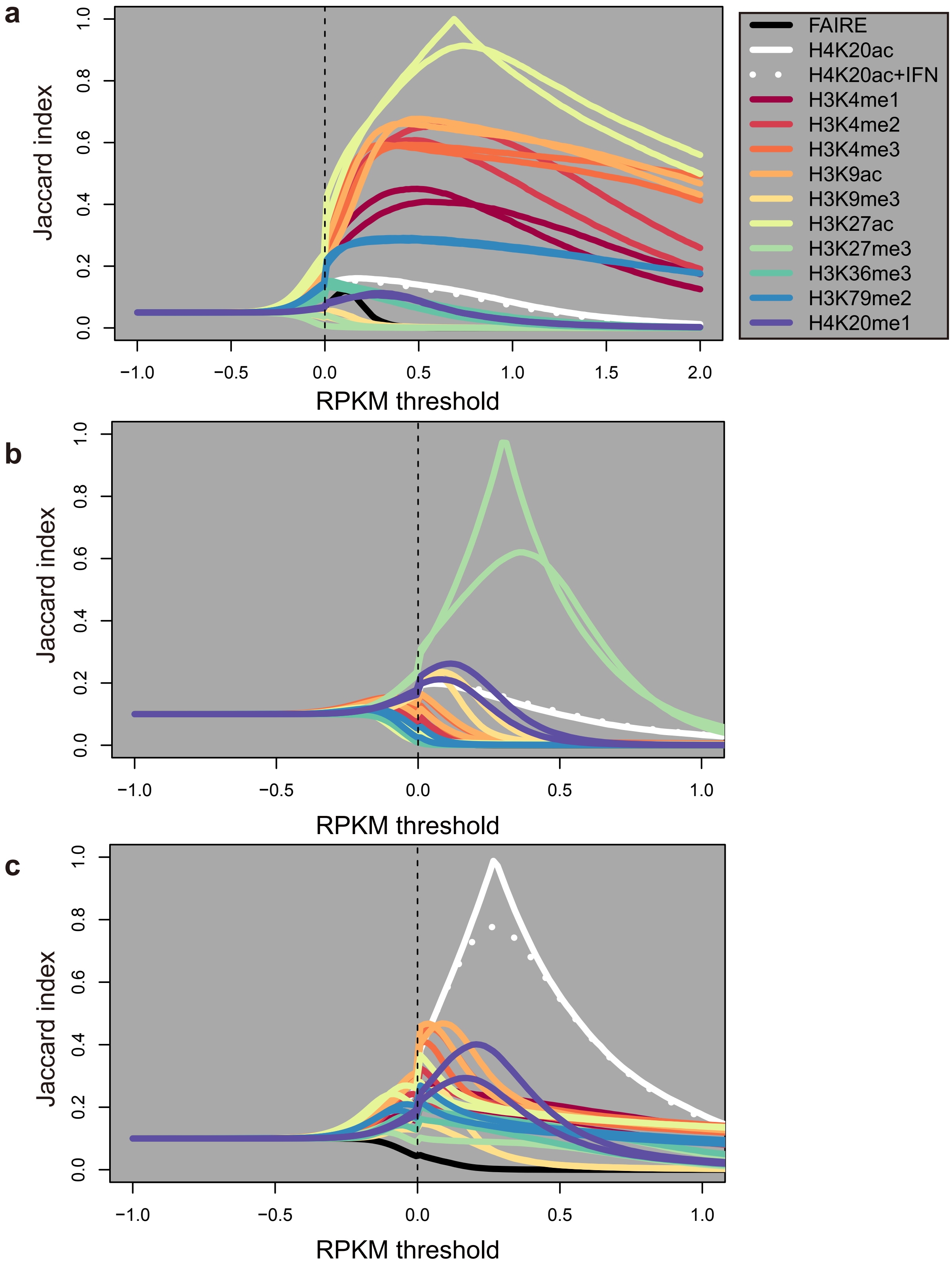

Supplementary Figure S3-2. Colocalization analysis: Jaccard index curves.
Jaccard index curves were plotted as functions of the cutoff levels. ChIP-seq gene sets positive for H3K27ac (a), H3K27me3 (b), and H4K20ac (c) were compared with other histone modifications indicated in the upper right column. For some modifications, two curves with the same colors are shown since two independent ENCODE datasets (rep 1 and rep 2) were available.
The Jaccard index, $J \in [0,1]$, measures the similarity between two sets, A and B, calculated as $\#(A \cap B)/\#(A \cup B)$. If the sets A and B were regarded as true and positive labels respectively, J is also calculated as $\#TP/(\#true + \#FP)$. For example, $J = 1$ if all the positive labels completely match all the true labels of a matching control ($A = B$), and $J = 0$ if all the positive labels were on the false labels ($\#(A \cap B) = 0$). Therefore, higher index values show higher overlapping.

Supplementary Figure S4. Transcription factor binding sites in H4K20ac-enriched sequences.
Aggregation curves of H4K20ac enrichment across the binding motif site of indicated transcription factors on the human genome (upper panel) and across the ChIP-seq peak of transcription factors (lower panel) are shown. USF, E2F and NFY_Q6 in aggregation curve data represent all of each factor isotypes or all factor subunits.

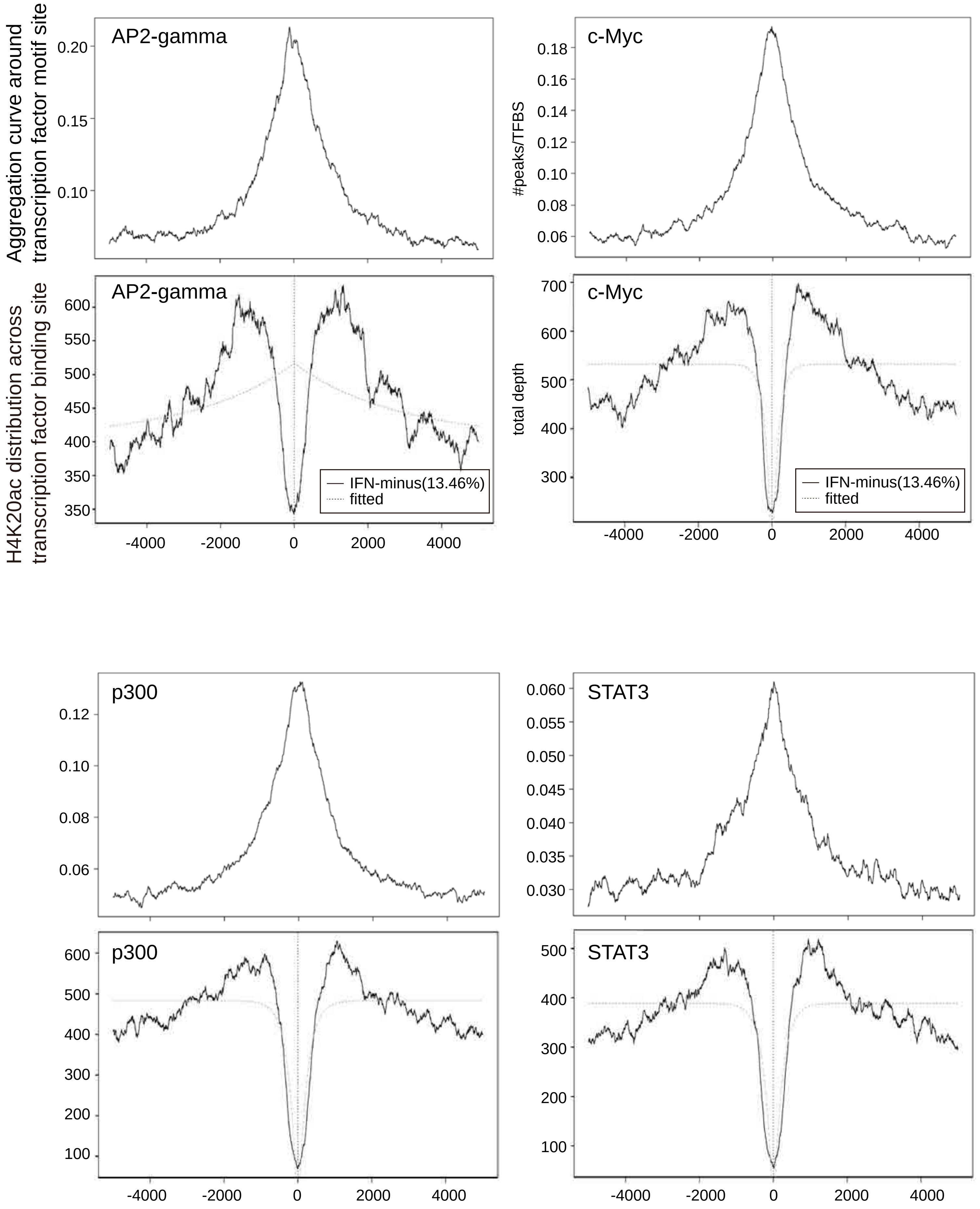

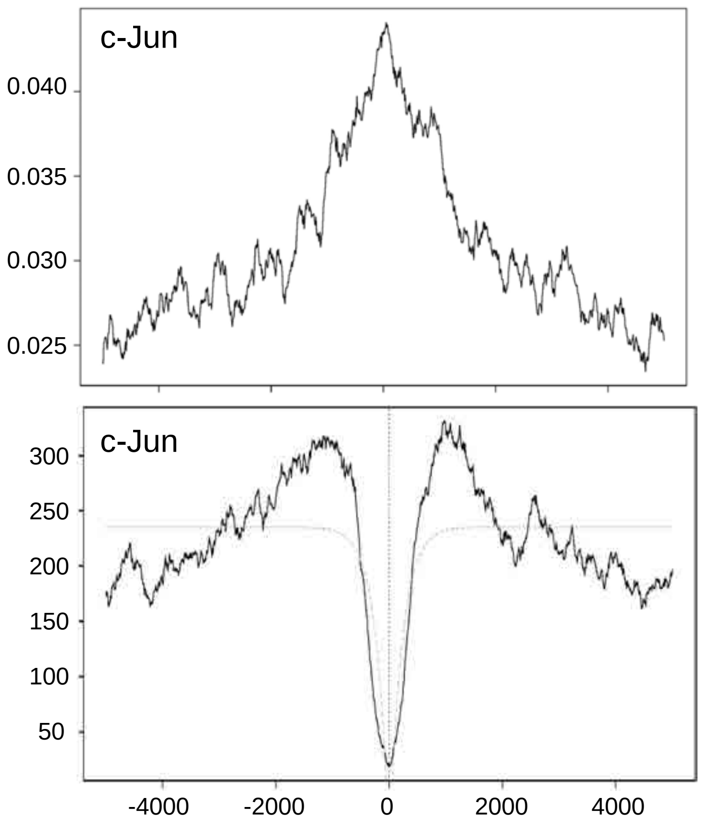
c-Jun
0.040
0.035
0.030
0.025
c-Jun
300
250
200
150
100
50
-4000
-2000
0
2000
4000

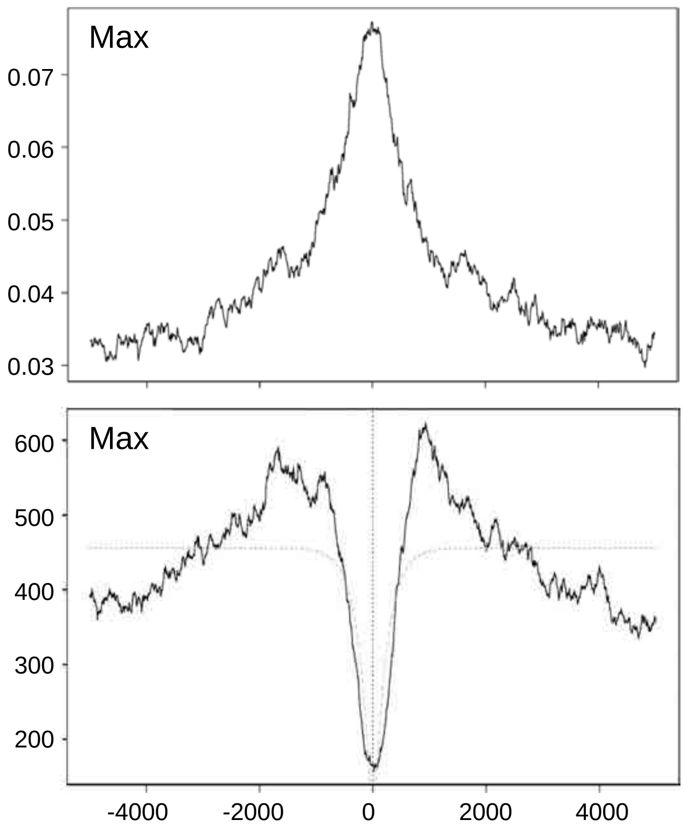
Max
0.07
0.06
0.05
0.04
0.03
Max
600
500
400
300
200
-4000
-2000
0
2000
4000

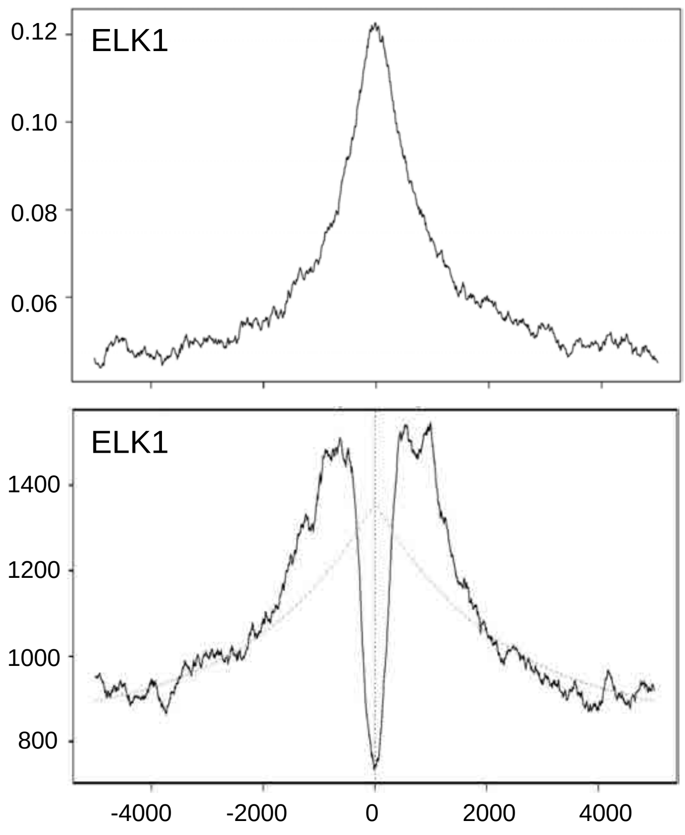
ELK1
0.12
0.10
0.08
0.06
ELK1
1400
1200
1000
800
-4000
-2000
0
2000
4000

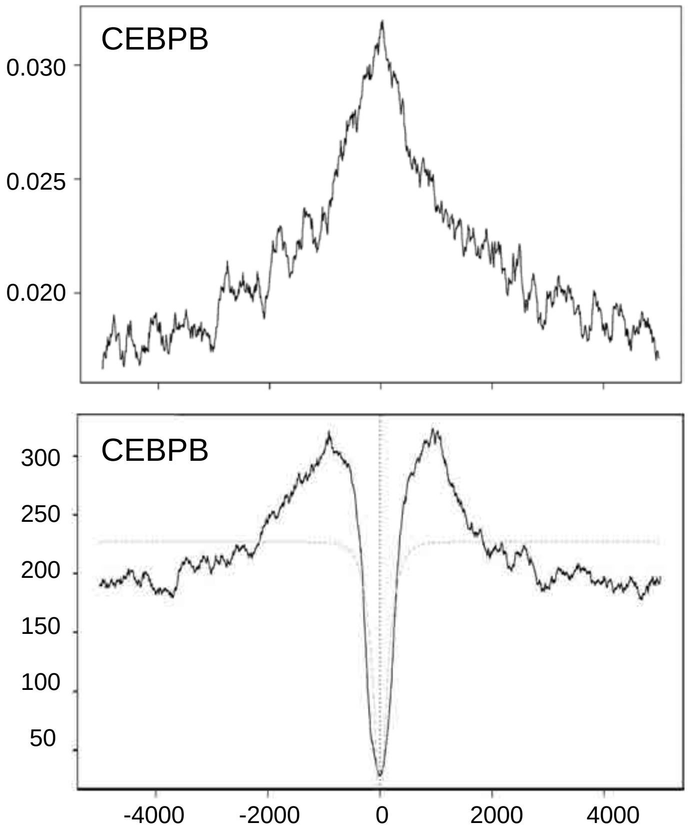
CEBPB
0.030
0.025
0.020
CEBPB
300
250
200
150
100
50
-4000
-2000
0
2000
4000

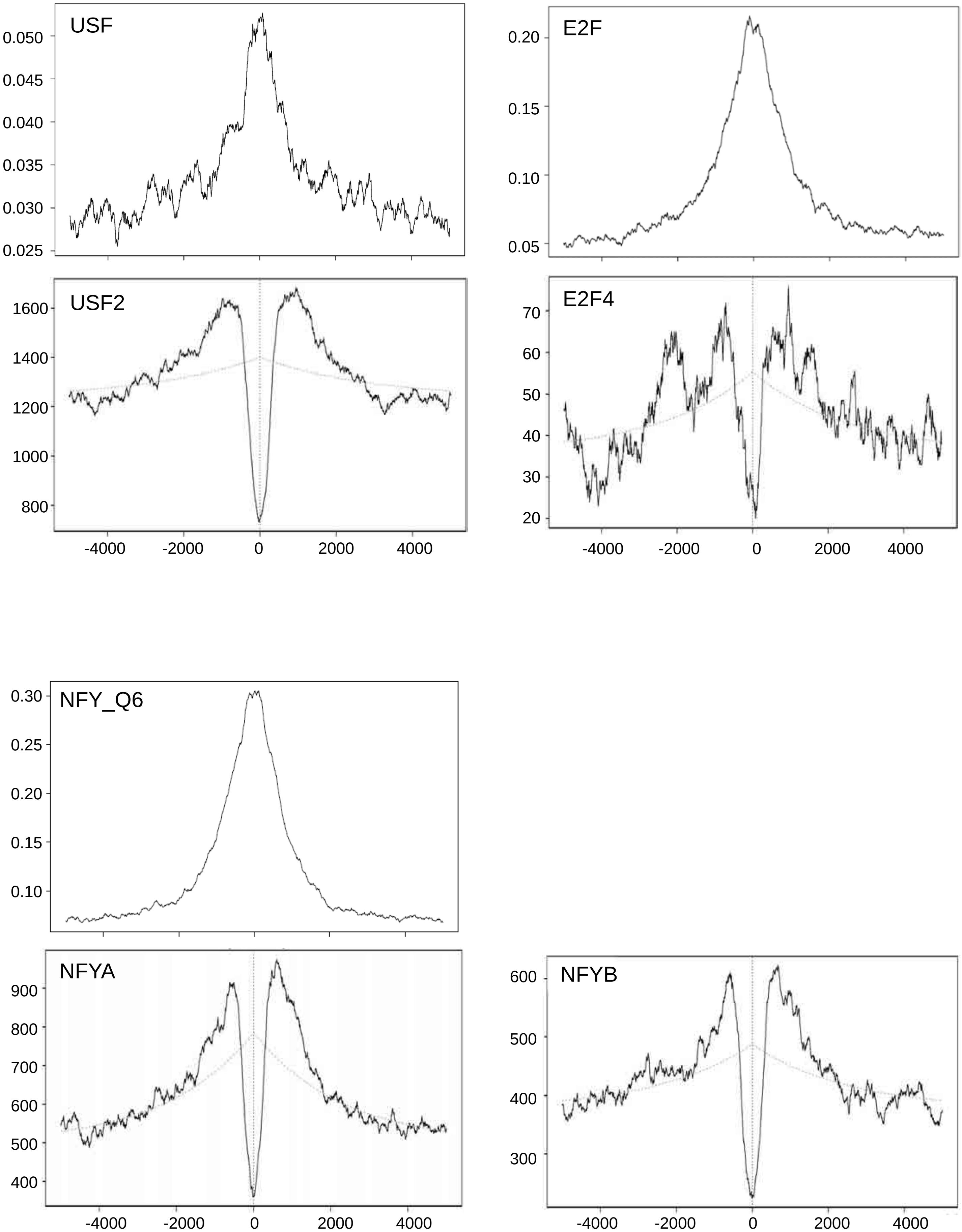
USF
0.050
0.045
0.040
0.035
0.030
0.025
E2F
0.20
0.15
0.10
0.05
USF2
1600
1400
1200
1000
800
E2F4
70
60
50
40
30
20
-4000
-2000
0
2000
4000
NFY_Q6
0.30
0.25
0.20
0.15
0.10
NFYA
900
800
700
600
500
400
NFYB
600
500
400
300

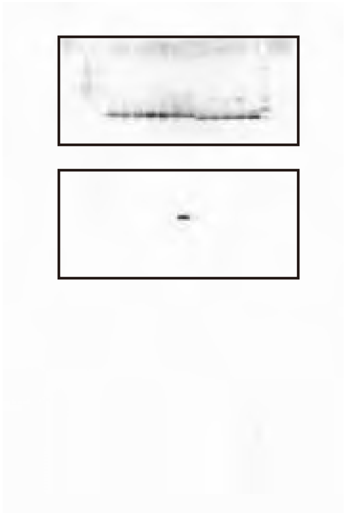

Fig.1 e,pan-H4(upper), H4K20ac(lower)

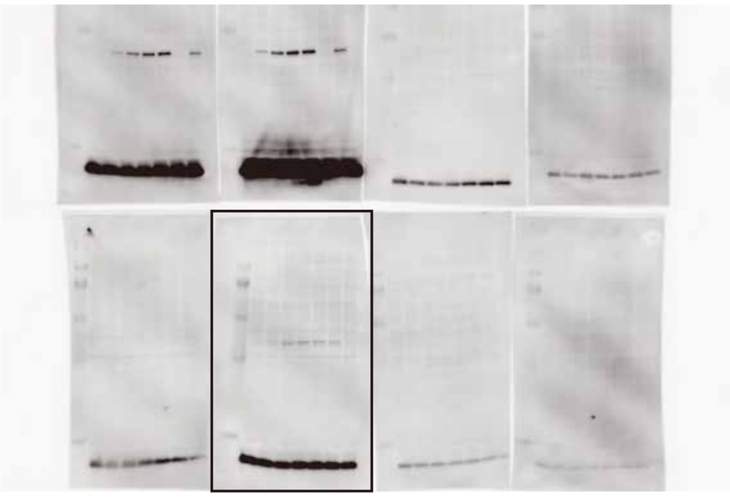

Fig.1 f,H4K20ac

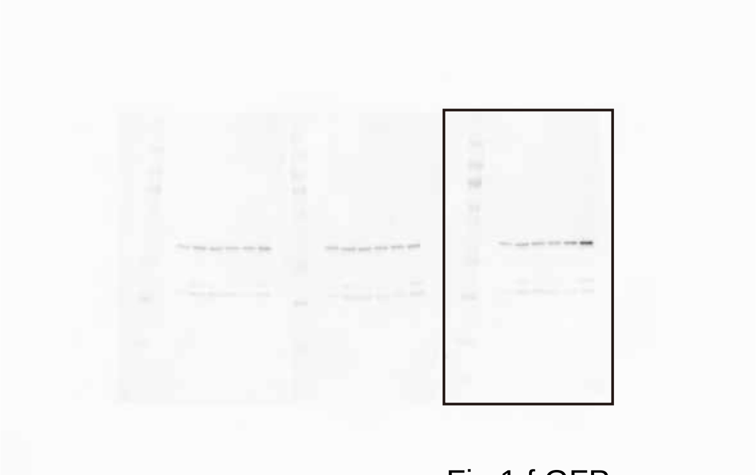

Fig.1 f,GFP

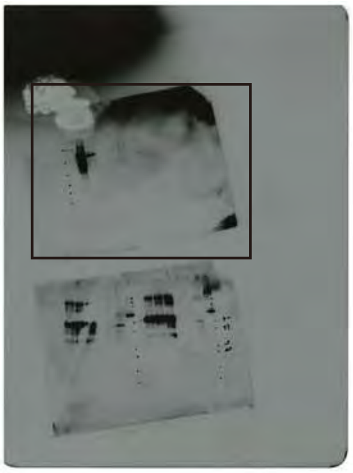

Fig.4 c, NRSF

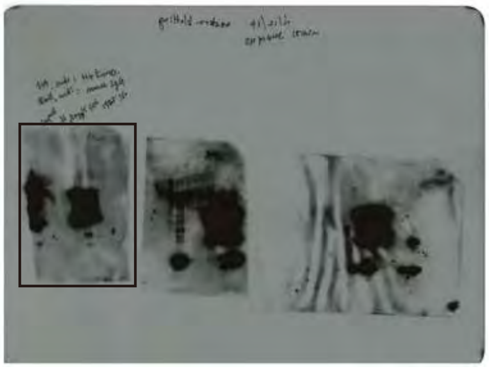

Fig.4 c, H4K20ac

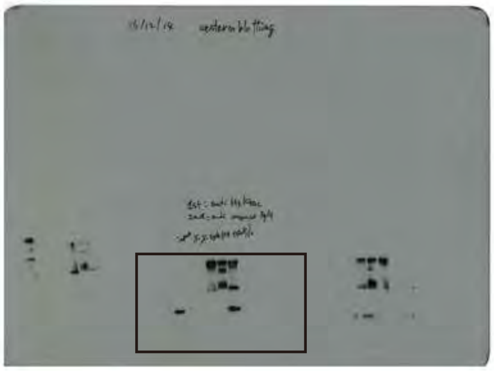

Fig.4 c, H3

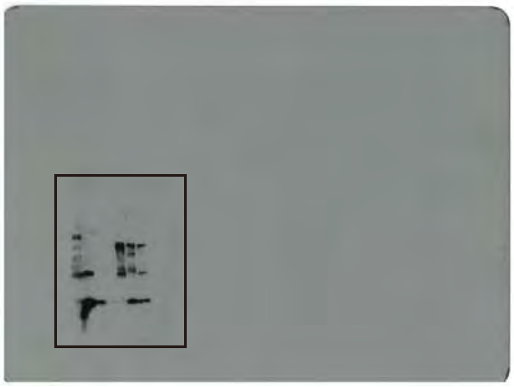

Fig.4 c, H4

Supplementary Figure S5. Complete immunoblots.
The original immunoblot data that were acquired by LAS3000 Imager (Fig. 1) and by X-ray films (Fig. 4) are shown. Sections shown in the main figures are highlighted in boxes.
